# Difference and subordination – the epistemic struggles of collaborative knowledge production in the field of mental health

**DOI:** 10.1186/s40900-025-00720-4

**Published:** 2025-05-13

**Authors:** Jenny Ziegenhagen, Ute Maria Krämer, Georgia Fehler, Guillermo Ruiz Perez, Daniela Schmidt, Lauren Cubellis, Madeleine Küsel, Sebastian von Peter

**Affiliations:** 1 Medical School Brandenburg Theodor Fontane, Fehrbelliner Strasse 68, Neuruppin, 16816 Germany; 2 Kellerkinder e.V. Germany, Berlin, Germany

**Keywords:** Coproduction, Code formation, Appropriation, Cooptation, User-led, Survivor, Qualitative research

## Abstract

**Background:**

Collaborative or co-productive approaches in the field of mental health care research are often legitimized by the argument that researchers with lived experience of mental health crisis and disability (= LE) produce different knowledge as compared to those without these experiences At the same time, there is a lack of studies that report on the underlying collaborative processes and on how these processes affect the knowledge that is being produced. This manuscript describes a collaborative research process and how this process impacted the knowledge produced.

**Methods:**

The collaborative research process entailed a multi-step coding process, using a variant of thematic analysis. To facilitate comparison, two code systems were produced, one by researchers with and the other by researchers without LE of mental health crisis and disability. Subsequently, the code systems of these two sub-teams were integrated into a single code system. To evaluate the potential differences between the code formations of the two sub-teams as well as the effects of their integration, three focus groups suceeded, composed of 1) psychology students as well as researchers 2) with and 3) without LE, whose results are at the core of this manuscript.

**Results:**

The focus group participants described extensive differences between the code formation of the researchers with and without LE – first in form, but also more substantially in the contents of both systems – corresponding to two distinct logics for understanding the implementation of PSW: an “institutional” and “interactional” logic. The integration process of both code systems was described as invasive, resulting in a final code system that more closely resembled the primary code system of the researchers without LE.

**Conclusion:**

The distinct logic of the two code systems can be thought of as distinct but complementary positions on the topic of PSW implementation. Such an explanation, however, falls short, as it silences the power relations and diverging interests and positions of the researchers involved. This is supported by what resulted from the integration of both code systems, resulting in the continuation of the logic of the researchers without LE. It is concluded that epistemic struggles and their knowledge politics require greater attention in the context of collaborative mental health research.

**Supplementary Information:**

The online version contains supplementary material available at 10.1186/s40900-025-00720-4.

## Abstract (plain language summary)

Collaborative or co-productive mental health research is often justified with the argument that researchers with lived experience produce different knowledge as compared to those without these experiences. This paper engages two research questions: 1) do researchers with and without lived experiences of mental health crisis and disability produce different types of knowledge in qualitative research? and 2) do these knowledges change through their collaboration? The paper reports on the processes of a collaborative research project on the implementation of peer support work in mental health care. We show that the perspectives and positions of researchers both with and without lived experience differed substantially, resulting in two distinct bodies of knowledge. When bringing these bodies together, we discovered that the perspectives of the researchers with lived experiences were dominated by those without. We conclude that power relations and divergent interests within collaborative research teams need greater attention in the context of co-productive mental health research.

**Supplementary Information:**

The online version contains supplementary material available at 10.1186/s40900-025-00720-4.

## Introduction

In the field of mental health research, collaborative or co-productive approaches are frequently justified or legitimized by the argument that researchers with lived experience as users of mental health care services produce different knowledge as compared to those without these experiences. These arguments are substantiated by empirical data. For example, the qualitative work by Gillard et al. demonstrates that researchers with lived experiences produced “analytical narratives” that addressed psychiatric violence and medication more strongly than the code systems of the other researchers that collaborated with them [1]. Similarly, Sweeney et al. used a collaboratively implemented thematic analysis and made clear that this collaboration not only led to *more*, but also to *new* thematic codes, particularly those that mapped the specific experiences and needs of users and survivors [2]. In our own work, the same set of transcripts was evaluated, first, by a team of researchers without lived experiences and, second, in a collaborative team. The second analysis concretized the specific perspectives of user/survivor knowledge, while the first coding process focused more on organizational and structure-related concerns [3, 4].

Thus, the impact of collaborative research seems to be well-substantiated. At the same time, there is a lack of studies that report on the underlying collaborative processes and on how these processes affect the knowledge that is being produced. Aveling et al. [5] frames the cooperation between institutions and actors that differ in status, resources, interests and needs as “ongoing practices” (ibid), meandering between “the ideal and pragmatic” (ibid.), thus requiring continuous compromise formation as well as endiuring dialogue and processes of negotiation. They conclude that more research is needed that examines the dynamics of these situated partnerships in interaction. Levitt [6] advises to better understand, how qualitative knowledge production is shaped by systemic power, oppression, and diverging forms of privilege, requiring a high degree of reflexivity to also contextualize its findings. In relation to the field of mental health, the survivor researcher Diana Rose [7] describes how wider institutional and political contexts and related hierarchies of knowledge and methods restrict the “hermeneutic space” (p. 139) of collaborative research with severe impacts on its results (Ref). In the same way, Lambert and Carr [8] describe how power and control are inherent to collaborative research processes and how they directly and indirectly mold the output of such studies. Our own work [9], such as the work by others [10] make clear that tensions, unsettling relationships and conflicts in collaborative teams can be both challenging and epistemically productive as they allow for changes in perspectives and positions of all team members.

Adding to these insights, this manuscript reports on the methodological procedures and resulting outcomes of a qualitative sub-study of the collaborative, mixed methods ImpPeer-Psy5 project, which explored the implementation of peer support work (= PSW) in the German health care context. As elaborated below, PSW designates the implementation of staff in mental health care services that bring their lived experiences of mental crisis, psychiatric violence, and recovery (= LE) to their professional role. The collaborative staffing of the research team intended to do justice to the different perspectives on these implementation processes in clinical practices, some studies describing these processes as absolutely necessary [11], while others – and here in particular from researchers with LE - being more critical [12–15], i.e. with regard to the negative effects on the PSW. On the level of our research team,, “collaboration” meant the close and paid cooperation during all phases of the study between researchers with and without LE, as further elaborated below.

This manuscript reports on our collaborative team processes, also describing how these processes impacted the knowledge produced. In the methods section, the collaborative procedures are described in detail, followed by a description of three succeeding focus group assessments, by which the types of knowledge emerging from thes procedures were evaluated, and whose results are at the center of this manuscript. The “Results” section presents the differences within the knowledge collaboratively produced and what may be lost if teams members with and without LE work together. The discussion contextualizes these findings with literature on epstemic agency, power dynamics and the politics of knowledge production. Thereby, the paper follows the research questionss of 1) which differential types of knowledge may be produced between researchers with and without LE in collaborative research teams, and 2) how these types of knowlegdes may be altered through the processes of collaborations? The ovreaching goal is to both sensitize the readers to the impact of collaborative research in the field of mental health and to exemplify some of its epstemic struggles and other fundamental procedural challenges.

## Materials and methods

### Project context

For more than 30 years, individuals with LE of mental health crisis, psychiatric violence, and recovery have been involved internationally in the provision of psychosocial and psychiatric health services as peer support workers (PSWs). Numerous publications discuss the challenges of implementing peer support work (PSW) in psychiatric and other care contexts [12, 16–21]. In Germany, questions around implementation processes have come into focus in the mental health hospital sector due to a personnel ordinance passed in 2016 (the PPP-RL law). As a result, core tasks and roles for this new professional group had to be defined and guidelines for their remuneration negotiated.

In this context, the ImpPeer-Psy5 study aimed to assess the current implementation status of PSW in the mental health hospital sector across Germany [22]. Second, the concerns of the stakeholder groups involved in this implementation (PSWs, other employees, and service users) were evaluated to derive concrete strategies from their experiences and requirements. To this end, a mixed-method QUAL-QUAN-QUAL design was implemented: Two qualitative research phases (QUAL 1 and QUAL 2) framed a standardized survey (QUAN) to investigate the existence and needs of PSW in nationwide hospital care. This was supplemented by a systematic review and a series of theory-of-change workshops (ToC) to develop strategies for successful implementation in the German health care system. The study parts were implemented by different teams: 1) a team at the Brandenburg Medical School (MHB) conducting both QUAL study parts, 2) a team at the University Medical Center Hamburg-Eppendorf being responsible for the QUAN, the ToC and the REV study parts, and 3) a team at the non-profit PSW training organization “EX-IN Germany” contributing as a practice partner in various steps of the research (for an overview of overall design of the ImpPeer-Psy5 study see E1 and [22]).

**Fig. 1:**
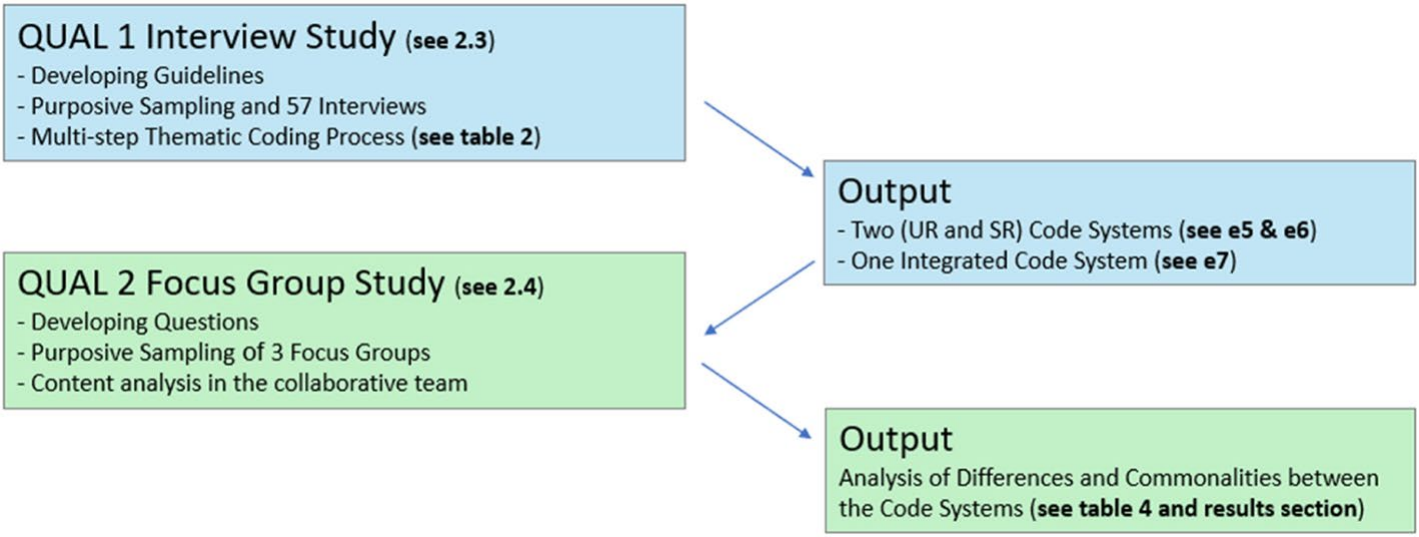
The interrelated processes of the QUAL1 interview and the QUAL2 focus group assessments

The present manuscript referes to material from both the QUAL 1 and QUAl 2 research phase of the ImpPeer-Psy5 study, both implemented by the MHB research team. On a more general leve, The QUAL 2 assessments aimed at further exploring further questions and inconsistencies that had arisen from the earlier study parts, and in particular from the QUAL 1 phase. For this purpose, the MHB research team was split into several sub-teams to host 10 sub-projects that explored a diversity of new questions, one – this one – of them focusing on the differential knowledge production of the researchers with and without LE in the MHB research team. For this purpose, a focus group sub-study took place (marked in green, Fig. 1) that analyzed material of the large-scale interview study of the QUAL 1 phase (marked in blue, Fig. 1), which is why this part of the study is described in the following first, i.e. in the chronologically correct order. At the same time, this description of the QUAL 1 proceedings is only to contextualize the findings of the QUAL 2 focus group study, which is at the center of this manuscript and its “Results” section (for more guidance on interrelated procedures see Fig. 1).

### Research approach

The ImpPeer-Psy5 study used a collaborative research approach combined with participatory methods and strategies to engage different stakeholders at different stages of the research process. Researchers and participants with and without LE of mental health crisis and psychiatric violence collaborated closely in all phases of the study: to develop the research proposal and questions, during the collection, evaluation and interpretation of the data, and during the publication of the results. Collaborative research approaches in the field of mental health are also referred to as co-productive [23], both terms being used interchangeably. Even though they differ from user/survivor-led or -controlled research approaches [24, 25], they may, as in our case, draw on the latter by following some of their principles or values, such as striving at equal partnerships between the research partners [26, 27].

In this manuscript, we understand the collaborative knowledge production as an inherently social process, thereby following various authors both from the fields of social sciences [28, 29] as well as from mental health user/survivor-led research [23, 24]. Such understanding conceptualizes research as emerging from situated encounters between the researchers and other participants involved, positioning the knowledge produced in a spectrum of institutional, political, cultural, and individual influences, such das the researchers’ differences in socio-economic status, positionalities, interests, values, and needs. The same is true for the understanding of lieved experiences or experiential knowledge: following various conceptualizations [7, 25], this is understood as collective systems of understanding and meaning that derive from shared experiences (of marginalization, psychiatric care, social deprivation etc.) and activities (of self-advocacy, PSW, self-help etc.) Thus, usual claims or assumptions of any objectivity of knowledge are renounced in this paper in favor of a more constructivist and post-positivist epistemology that understands knowledge (production) as inherently situated, rooted in specific experiences, and therefore perspectival.

To transfer this understanding of science to our project, more information is needed on the researchers that had been involved in the QUAL 1 interview study: All members of our collaborative team were formally employed at the MHB as researchers, drawing on different professional, academic, and experiential backgrounds. Further, they occupied different positions towards the mental health care system by having used/survived or having been employed as staff in mental health services, by having used or practiced PSW, or by having participated in related self-advocacy or political work. Crucially, they came from diverse disciplinary fields such as mental health service research, user-led/survivor research, medicine, anthropology, nursing, cultural studies, sustainability studies, religious studies, philosophy, religious studies, making our study also a decidedly interdisciplinary one (for a researchers-related overview, see Table 1).

**Table 1:** Disciplinary, professional, and experiential backgrounds of the researchers of the collaborative research team of the QUAL 1 interview study

**Researcher**	**Backgrounds**
UR 1	Peer Support Work, Social Anthropolgy, User/Survivor Research, Self-Advocacy and -Help
UR 2	Religious Studies, Collaborative Research, Self-Advocacy and -Help
UR 3	Peer Support Work, Collaborative Research, Self-Advocacy and -Help
UR 4	Peer Support Work, Self-Advocacy, Sustainability Studies
SR 1	Psychiatrist, Philosophy, Work alongside PSW
SR 2	Mental Health Nurse, Social Anthropology, Political Work
SR 3	Psychiatrist, Social Anthropology, Work alongside PSW, Research in PSW, Political Work
SR 4	Social Anthropolohy, Research on PSW, Political Work

To navigate this complexity, the terms (mental health care) “user researcher” (= UR) and (mental health care) “staff researcher” (= SR) are used in the following. This distinction certainly simplifies the many other differences between the members of our collaborative research team, such as differences of age, gender, education, sexual orientation etc.. Yet, given the complexity of the research process and analyses, and the rather minimal resources for undertaking it, an intersectional analysis was not possible in this project, even though it is of major importance during collaborative knowledge production [30]. Furthermore, both these backgrounds – of having either used mental health services or of having worked in these institutions as a staff member without any LE – were central to the understanding of our research topics: 1) the implementation of PSW an 2) the varying perspectives on this topic from members of the collaborative research team.

As stated and shown in Fig. 1, this manuscripts discusses the differential knowledge production during the ImpPeer-Psy5 QUAL1 research phase. In this phase and during the QUAl 2 phase, including the preparation of this manuscript, the MHB team consisted of four UR and four SR, who worked individually, in small groups, and as an entire research team in different steps of this research phase (see Fig. 1, “The QUAL 1 interview study” section, and Table 1). To report on these procedures, the GRIPP-Checklist was used, which is presented in the Electronic annex (E2).

As described above, in the following, the QUAL1 assessments of the ImpPeer-Psy5 study are detailed first, whose results that lay the ground for the subsequent focus group study, evaluating the types of knowledge produced during the these assessments.

### The QUAL 1 interview study

#### Sample and recruitment

The participants for the QUAL1 interview study came from three stakeholder groups: 1) PSWs who are or have been active in providing mental health care in different health care sectors, 2) (former) user of PSW from these sectors, and 3) employees from other professional groups that (have) work(ed) together with PSWs, i.e. trained in psychology, social work or nursing, as well as staff from the management level. This sampling approach was intended to generate a multi-perspectival body of knowledge concerning the requirements and challenges of implementing PSW in psychiatric care.

Recruitment took place during study months 6 through 30, which spanned the years 2021-2023. The training network, EX-IN Germany, and smaller training organizations (EUTB, Lebensart Münster, UPSIDES, etc.) contributed as multipliers. In addition, information on the study was shared on PSW-related social media platforms to facilitate the recruitment of study participants. A total of 57 participants (32 PSW, 19 other employees and 6 users) were recruited for the QUAL1 interview study, using a combination of a snowball sampling and purposive case selection. Overall, the participants were selected from all 16 federal states; from rural, urban, and metropolitan areas; from northern, southern, eastern and western Germany; as well as from different treatment settings (inpatient, day care, outpatient, home treatment). The socio-demographic composition of the population can be found in the Appendix (E3).

#### Guideline development and assessments

To develop the interview guidelines for the QUAL1 phase, the MHB team divided into small groups, which generated a total of approximately 280 potential questions based on their different knowledge and experiential backgrounds. These questions were systematically sorted in several steps and evaluated in relation to domains from the literature. This process was time-consuming and required close coordination between all researchers. The dialogues were often intense due to the different backgrounds and personal/professional positions outlined above.

The negotiation resulted in a total of 76 interview questions, which spanned themes such as PSWs’ trainings and competencies, institutional requirements prior to their implementation and institutional onboarding, PSWs’ tasks, and roles, interprofessional collaboration, strategy and mission statements, possibilities for the PSWs’ participation on an institutional level, policy development, cooptation etc. (for more details on the overarching research questions see E4). These questions were separated into six interview guides (three for PSWs, two for other staff, and one for service users, with ten participants recruited for each guide = 30 PSW, 20 staff, 10 users). The interview guides were discussed in three participatory focus groups with a total of 29 stakeholders from various backgrounds. This resulted in the modification of many individual questions as well as whole groups of questions.

To enable a safe interview situation, the interviews were conducted in pairs, ideally by one UR and one SR. The interviews were recorded and anonymized during transcription.

#### Analysis and evaluation

After transcription, a multi-step coding process was implemented by the MHB collaborative team (see Table 2). Following the coding approaches of comparable projects [1, 2], a variant of thematic analysis [31] was used, which was adapted to a collaborative research design but involved the six steps of analyses described. In the first step, all team members read the transcripts in detail. In step 2, a training was held to familiarize the team members, who had very different orientations to coding processes due to their different disciplinary backgrounds (see “Research approach”), with the planned procedure. Parts of the transcripts were then coded by the entire team to build consensus on the length and type of coding units to be used. Third (see Table 1), the transcripts were divided evenly among the team members to enable the individual and inductive coding of all transcripts by two team members each. All transcripts were coded by one UR and one SR, respectively. These inductive codes were gradually integrated into two code systems with the two subgroups of UR and SR working in parallel (step 4). During this process, overarching categories were developed that integrated the single codes produced in the inductive coding process. This integration required intensive collaboration between the group members to iteratively identify, validate, extend, and structure the themes and categories. This can be considered the main analytical work of both sub-groups: defining the focus and negotiating the prioritization of both codes systems (see E5 and E6 in Electronic annex).

**Table 2:** Multi-step, collaborative coding process of the QUAl 1 interview study

**Nr.**	**Research Activities**	**Participants**	**Outcomes/Outputs**
1	Recurrent reading of the transcripts by the full research team	Full research team	Familiarization of the material
2	Coding-as-a-trial in the full team to negotiate the scope of coding sections and depth	Full research team	Clarity on coding sections and depth
3	Inductive coding of each transcript by two team members, one UR and SR	One single UR and one single SR per transcript	Inductive codes of transcripts
4	Structuring the codes into two, overarching code systems, one UR system and one SR system	One group of UR and one group of SR	Two overarching code system, one UR and one SR system
5	Approval of both code systems on basis of the inductive coding (step 3)	One group of UR and one group of SR	Approved code systems within both sub-teams
6	Comparison of the two code systems in the full research team in a full-day workshop	Full research team	Initial interpretation of differences between systems
7	Gradual merging of the two code systems into one system by comparing and adapting them	Groups sessions with a variable composition of UR and SR	One integrated code system
8	Approval of this system in the full team on basis of the inductive coding in step 3	Full research team	Approved integrated code system
9	Explication of code definitions and anchor citations	Full research team	Defined code system
10	Re-coding by two team members	One UR and one SR	Coded transcripts

The aim of implementing this parallel integration process was to facilitate the systematic comparison of both code systems further on in the process (see steps 6 and “The QUAL 2 focus group study”). Thus, after their approval by the subgroups (step 5), the two code systems were compared with each other in the full MHB research team during a full-day workshop to understand more about their similarities and differences.

After this comparison, the code systems were merged into a single code system through a multi-step process, involving both UR and SR and being coordinated by one of the UR (DS) (see E7 = integrated code system): initially, two synopses were planned, one integrating the codes and categories based on the UR system and the other integrating the codes and categories based on the SR code system. This two-sided approach was designed to prevent the dominance of one of the two systems over the other and to enable a bidirectional integration process. However, due to sickness, absences, and other reasons, which are described in the discussion, only one synopsis was developed (Table 3 depicts an excerpt from it). In this synopsis, the two code systems were integrated with each other in five small groups sessions staffed in shifts by both UR and SR, with one UR (DS) being continuously present to ensure the continuity of this work. To facilitate this work, the integration was carried out primarily at the level of individual codes and only in some cases at the level of overarching categories. For various reasons, which are addressed in the discussion, it was decided to use the SR system as the central basis. This resulted in a significant subordination of most UR codes to the categories of the SR system. There were exceptions to this unidirectionality but for the most part, the SR category system guided the integration process. Again, this was a time-consuming process that required extensive reflection on word choice and debates about shared meanings, all while aiming to avoid hierarchical decision-making processes.

**Table 3:** Excerpts from the synopsis that served the stepwise integration process of both code systems of the QUAL 1 interview study

Categories SR	Codes Level I SR	Codes Level II SR	*Code Level II UR*	*Codes Level I UR*	*Categories UR*
Experiences and qualifications of PSW	Treatment experiences		*PSW know the everyday routines of ward*	*Know-how*	*Interactions between PSWs and users*
			*Similar problems despite distinct symptoms*		
			*Less prejudices due to own crises experiences*		
	Educational experiences and qualifications		*Additional qualifications and work experiences*	*Education*	*Peers Only*
Preparation of institution	(No) preparatory measures		*Institutional preparation (for instance job description and interview)*		*Structures/Processes*
	No preparation for challenges of collaboration				
	Non-useful preparatory measures				
	Teambuilding measures bevor implementation				

The resulting code system was explicated by code definitions and anchor citations, as well as checked for completeness and plausibility, by the entire MHB research team based on the first inductive code formation (steps 8 and 9). During step 10, this integrated code system was used to deductively re-code all transcripts by two team members, remaining in conversation with each other and with the entire MHB research team in case of uncertainties or questions.

### The QUAL 2 focus group study

As outlined, this multi-step, complex coding process was implemented to facilitate the comparison of potentially divergent perspectives and bodies of knowledge among the team members. As described in the introduction, other researchers have identified such knowledge differences between UR and SR, which highlights the importance and impact of collaborative research approaches. Our approach builds on this work by making use of the polyphony and heterogeneity of perspectives and positions of our collaborative research team to ultimately increase the comprehensiveness of the final code system.

As described in step 5 under “The QUAL 1 interview study” section, the inductive code systems developed in the two subgroups were first compared at MHB team level. This took place during a full-day workshop between all members of the MHB research team, during which to plan further steps for integration. Significant differences between the two code systems were identified and documented. Moreover, it became clear that it was necessary to investigate these differences more systematically, after the analysis of the QUAL 1 material was complete.

To this end, three focus groups (FG) [32] were put together during the QUAL 2 research phase: a group of 11 students of the MHB psychology master’s program (“outsider perspectives” – FG 1), one group of five UR (with expertise in research, activism/self-advocacy and/or peer support work – FG 2) and a third group of five SR (with expertise in mental health service and social science research as well as working experiences as psychiatrists or psychologists – FG 3). The participants of the last two FG hat not been involved in the ImpPeer-Psy5 study previously, but did have experience working in other participatory-collaborative research projects (“insider perspectives”). A focus group approach was chosen to assemble the assessments of various experts who had not been part of the previous coding process or the research team. We had discussed a document analysis [33] as an alternative, but rejected this option because, given the complexity of the material, we aimed for a discursive and multi-perspectivist examination of the code systems. The implementation of three focus groups derived from the hypothesis that the perspectives on our material would differ according to the different backgrounds and positions - a hypothesis that was not confirmed, as explained further in the discussion.

The recruitment of participants was targeted, while attending to heterogeneous socio-demographic characteristics. To prepare the FG, all participants were sent 1) the UR code system, 2) the SR code system and 3) the integrated code system together with the study documents. They were asked to read these documents and prepare for the following tasks:

1. Please compare the code systems of both subgroups and consider the question, what differences do you see between the two systems and what similarities (type of codes, kind of language, arrangement/sorting of the codes, etc.)?
2. Examine the merged code system and consider the question: How do you experience the merging of both code systems? Which content has been dropped or neglected, what has come to the fore after this process?

The FG were led by two members of the MHB team with different professional, disciplinary and experiential backgrounds (SvP and JZ) and took place in persona in November and December 2022. The FG with the master’s students lasted four hours (with a pause), the FG with the participants of the other two groups lasted 90 minutes each. All FG were audio-recorded, transcribed and coded in parallel by SvP and JZ using an inductive content analysis [34]. From this, a code system was created (see Table 4) and discussed with the entire MHB research team.

**Table 4:** Code system of the QUAL 2 focus group assessments

Total number of codes	510
**Formal differences**	
Definitions	6
Sequence	5
Length	9
Language	33
Understandability	15
Redundancy	3
Comprehensiveness	4
Level of concreteness	4
Grade of neutrality	19
Closeness to citations	10
Perspectivism	32
Political dimension	32
**Differences in contents**	
Interaction logic	21
Closeness to experiences	8
Getting to the point	16
Emotionally laden	24
Closeness to life	18
Structures and human beings cannot be separated	9
Oriented at basic needs	9
Institution logic	8
Criticism of PSW	19
Talking about other	2
Distance	7
Legal and technical framework	40
Commodification	11
Reproduction clinical othering	1
Processes from people	3
**Impression on the readers**	
Structure/Sequence	2
Readability	11
Being able to empathise	9
Rousing	6
**Effects of integration**	
More organization	3
Neutralization of emotions	8
Loss of clarity	3
Complementarity/completion	9
Shifts and Changes	27
Shifts in contents	12
Loss of focus on relationships	5
Gain of focus on structures and processes	5
Loss of peer perspectives	13
**Imagined processes of integration**	
Assumed social processes	20
Possible alternatives	4
**Basic science-theoretical concerns**	10

This publication refers exclusively to material from this QUAL 2 FG study. It was prepared primarily by JZ (UR) and SvP (SR) in collaboration but was also discussed several times with the entire MHB research team.

## Results

The results of the QUAL 2 FG study (code system see Table 3) are subdivided along two chapters: first, the differences between the UR and SR code systems are presented, as identified by the FG participants (“Identified differences between the UR and SR code systems”); second, the participants’ perceptions regarding the integration of the two code systems are displayed (“Perceptions on the integration of the two systems”). The citations marked with “FG 1” refer to the students of the psychology master’s program, “FG 2” refers to the participating researchers with expertise in research, activism/self-advocacy and/or peer support work, and “FG 3” to the participants with expertise in mental health service and social science research as well as working experiences as psychiatrists or psychologists.

### Identified differences between the UR and SR code systems

The comparative statements of the three FG participants have been assigned to the four subsections presented below: 1) structure of the code systems, 2) language used, 3) focus of content, and 4) overall impression.

#### Structure

Both code systems differ in terms of their length and order of codes and themes. The UR code system is significantly shorter than the system of the SR. Further, the UR code system contained a sub-section that was called “peers-only”, attuned to codes that concerned PSWs exclusively. This was highlighted by the FG participants as well. FG comments on the order of the UR codes revealed a potentially exciting topic right from the start:

“What I noticed about the UR code system is that it starts with role conflicts. I found that interesting. This is the first code, I found that kind of exciting, to say, okay, we’ll start immediately with a role conflict. One could philosophize about what this might mean.” (FG_2)

The SR code system consisted of three parts, each referring to one group of participants in the QUAL1 survey (PSWs, other employees, and users). According to one FG participant, this structure also impacted the orientation of the codes:

“So, in the second [SR code system] there is more of this professionalization logic, e.g. the tripartite division along the various occupational groups; the other [UR code system] is more integrative-organic (...) in the second system [SR system], there are way more codes in the system that code for the employee transcripts, three times as many as in the first [UR] system.” (FG_3)

The SR code system used an institutional-sequential logic: the topic of training was coded for at the beginning, followed by codes that addressed the institutional preparatory processes and the PSWs’ hiring processes. Only after this did it turn to the specific tasks and roles of PSWs, as well as the topic of their collaboration with other employees, which in the eyes of the FG participants reflected a weighting of these topics:

“This sequential structure of the second code tree [= SR code system] follows an institutional process orientation: “ways in” is the first category, then “preparatory measures of the institutions”, which is notincluded in the first code tree [UR system], these themes are rather included in some subcategories that come later in the sequence.” (FG_3)

In this sequential logic, codes on the topic of interaction or conflicts between the employees of the different occupational groups only appear later in the code system of the SR:

“I counted (...) in the UR system, “interactions” appeared on page 1, which is the essential thing also for me when it comes to the question of implementing PSW. In the code system of the SR, this was only the case on page 11, so it came far later and was also assigned less subcodes.” (FG_2)

#### Language

The language of both code systems reveals different orientations of the two research groups. In the UR system, the FG participants noted the use of an everyday language and some “sociological terms”, whereas the language of the SR system was described more as academic and oriented towards standards of scientific work (“academy filter”):

“Academically appropriate language [in the SR system]. How can we translate what other people are saying in a way that sounds nice to academics? That’s what I had to think about all the time… “ (FG_1)

“… perhaps also the choice of words [...] I have the feeling that it is everyday words such as “freedom” and “openness” and “autonomy” that dominate in the peer tree.” (FG_3)

This bureaucratic-academic language caused some difficulties in understanding the SR code system. The language of the UR system, on the other hand, was perceived as more accessible:

“I have the feeling that the first [UR system] simply says what it means, it is easier to understand, more accessible to many people. Barrier-free, as barrier-free as possible…academic language is not barrier-free.” (FG_1)

Further, it was expressed that the SR code system used less often the terms and notions used by the interviewees themselves (in vivo codes) in comparison to the UR system. Additionally, a different degree of abstraction was found, with the FG participants perceiving a technical-factual orientation of the SR language, whereas the language of the UR was recognized as more emotional:

“… with the UR, I think the emotional part is much more weighted and much more central. You notice that immediately when you read it.” (FG_1)

These differences were exemplified in a discussion of the term “authenticity”. This term was not used at all in the UR code system (in contrast to the SR code system), but the codes *themselves* were perceived to be “more authentic” or to “express authenticity”:

“… I was thinking about the word authenticity all the time and the funny thing is that group 1 [UR] doesn’t even use this word, but group 2 does. I found that very significant and super interesting, because some people theoretically think about it and others actively transport it in their codes.” (FG_1)

In addition, the language of the SR was described by individual FG participants as “more clinical” and “top-down”:

“’Promote destigmatization.’ I would say that this is a classical science-professionals’ terminology. And “encouraging self-efficacy” is psychotherapists’ language.” (FG_3)

One of the participants recognized the structure and language of the SR code system to be othering:

“…in the SR system, as it is always the case, the deviants are observed and not those who are the norm, and the norm are the non-crazy ones and they are not observed at all, but the crazy ones are those who now come to the system as a new workforce.” (FG_2)

What was interesting here was the perception of one UR participant that the SR code system treated experiential knowledge as a commodity, which seems to also contribute to this distancing:

“…what I found interesting was that experience [in the SR code system] was treated like a commodity, i.e. experience independent from people. They talk about “experience” and what this experience brings to the table, and not what the people do, who bring this experience with them. I found that super interesting, as if the experience was a thing that you can push from left to right, a factor independent from people who have lived through these experiences…” (FG_2)

“For me, this is a kind of hidden devaluation. That’s very interpretative now, but for me it has definitely a distancing effect. If you reduce a human being’s part to a variable, it’s always strange, and if you reduce experiences to a variable, there is something diminutive about it.” (FG_2)

#### Focus of contents

The FG participants identified two different implementation logics. The SR code system was perceived to focus primarily on the organizational side of the PSW-implementation. According to their perceptions, it reflected the SR efforts to map the necessary conditions and steps of implementation mainly in relation to structural and institutional aspects:

“… the [SR code system] focusses more on the structure around it, what does the structure have to offer to the implementation, so to speak.” (FG_1)

As a result, the SR was seen as focusing on how the new professional group of the PSWs could be “organized into” the existing working environment of mental health care:

“…more this question: how do I get the PSWs better into the team structure or, what can I do to make them compatible with this structure?” (FG_3)

The UR code system, on the other hand, was perceived by many FG participants to follow a logic that understood the implementation of PSW as a social and interpersonal process:

“But with code system 1 [UR system], I would say that the implementation is more about the individual people and their relationships and interactions, in the sense of a social process.” (FG_3)

Thus, the UR system placed greater emphasis on the relationships between users and PSWs than the code system of the SR, drawing attention to the well-being of all stakeholders involved in the implementation of PSW:

“The first system [UR] is more about human experience, no matter who it is. And the second is about roles, somehow experience in roles.” (FG_3)

Overall, the UR code system was described by some participants as “more direct”, or “immediate”, in the sense of being more “at the core” of the implementation process:

“I think the first system [UR code system} was, as I said earlier, more directive, it was more emotional, more designed around the core of the implementation.” (FG_1)

This allowed some participants to get into the topic of PSW implementation more quickly and to understand more directly what it is all about:

“Code tree 1 [UR system] made me better understand what all is about, what is this profession of PSW, what they do and intend to do in mental health care”. (FG_1)

#### Overarching impression

The FG participants felt that the SR code system was “more value-free” and “politically correct”, but also more distant, which was explained by the fact that the SR were less directly affected by the topic:

“… of course, they are generally more distant from the whole topic, because it affects them as a research topic but not as a person. As a result, their codes are more value-free, perhaps also trying to keep their own opinion out of it, while that is not the approach of the first code tree [of the UR]. They don’t try to keep their own evaluation or their own opinion out of it.” (FG_1)

At the same time, restraining from values, this “keeping out of it” was described as only “supposedly neutral”, intended to underpin the “scientificity” of the SR code system, thereby also conveying a “sense of hierarchy”:

“...neutrality is the wrong word. For me, the second code tree [SR code system] came across as more value-free, yes, but also more emotionally distant…as if this perspective is the more “scientific” one. But it was not neutral either, rather positioned questions of PSW implementation on a different level, alongside a different perspective. This is not neutral. Thus, this for me is somehow a supposed neutrality, and, again, underlines this sense of hierarchy for me again. That bothers me.” (FG_1)

For some participants, the SR code system was understood as a view “from the outside”. It gave them the impression that the SR were not involved, rather taking a distant position on PSW:

“What you said so nicely earlier, they look at it from the outside and don’t go from the inside to the outside, but from the outside in.” (FG_2)

As a result, the SR system was understood to be problematizing the topic of PSW implementation compared to the system of the UR:

“The second [SR code system] is more critical of PSW, in the sense of: okay, this must be much more regulated, a lot is missing… all the positive things [of the UR code system] are appreciated too, but for the PSWs to be helpful, a lot of things must be organized. That’s why I perceived the second code tree to be a bit more negative, and the first one is very positive, very benevolent for the sake of PSW.” (FG_1)

One FG participant suspected that this problematization was due to greater uncertainty among the SR in relation to what is important for the implementation of PSW:

“In the first [UR system] a greater clarity and in the second [SR system] a greater uncertainty can be found [...] in the spirit of: don’t overlook anything [...]” (FG_1)

In contrast, the UR were perceived to be more certain about what is needed for this implementation, in part because they are more directly affected by the topic. They expressed themselves in a more demanding way:

“In the first [UR system], there are emotions behind it, there is a demand behind it: it shows that something must change […] whereas the second is more neutral…neutrality is not a bad thing.” (FG_1)

Other participants described the UR code system to “promote” the implementation of PSW:

“… I think it’s so fundamentally positive, according to the motto: it’s easy to implement it, and there are so many positive things, and we can do it.” (FG_1)

Other participants have experienced the UR code system as “confrontational” and “subversive”, containing demands for more fundamental changes in psychiatry, e.g. criticism of current treatment methods or the questioning of diagnoses. In this context, the UR were suspected of having a “psychiatric-critical” attitude and their code system was described as an expression of a “radical approach”:

“I think the first code tree has a very revolutionary character and is also there... I don’t want to say “over-motivated”, but very committed. Personally, I had the impression that the [PSWs] are against an existing system. It somehow makes it clear that they are trying to overturn something [...] the second code tree is more realistic, maybe that you can’t change the system completely, but that the PSWs must somehow integrate themselves into it to promote change.” (FG_1)

These divergences reveal that the starting points for change and transformation are understood to be differently located and conceptualized in the two code systems:

“I would like to ask both groups each, where do you think change happens? Because I believe that some believe that change happens on the structural level, and others believe that it happens in the interaction of the human.” (FG_2)

### Perceptions on the integration of the two systems

Discussing the effects of integrating the two codes systems, the FG participants made some assumptions about the underlying process within the research team that are summarized in the first section. In the second section, statements about the perceived effects of the integration of both code systems are discussed.

#### Assumed social processes behind the code systems’ integration

Different ideas were exchanged during the FG in relation to the social processes between the UR and SR. Some participants thought that the UR code system had been “incorporated” into the system of the SR:

“…some things were taken and exchanged, but actually it [the SR system] is very dominant; like a shelf that was already quite full. The UR version was sorted in and maybe one or the other time it was set higher or lower or maybe even replaced like a box, I don’t know.” (FG_2)

Others suspected a compromise solution between the two groups:

“There are fusion processes in collaborative research, if you bring perspectives into communication, into contact with each other, so to speak, and if you bring together perspectives, one is a bird’s eye view, and one is an insider perspective. But I don’t know how it went. Basically, I didn’t have a bad feeling when I saw that something integrated came out in the end.” (FG_3)

“I experience this code tree as the result of a discussion, yes. And the result of a discussion is at best a compromise.”. (FG_1)

There was speculation as to whether, during the process of merging the two systems, the SR group had the power to decide on which contents should be included. Still others described the integration as a necessary process to adapt the final code system to academic requirements:

“In this integrated code system, it seems as if the SR group has said: no, we must make the whole thing somehow more value-free, more academic.” (FG_1)

#### Perceived effects of the integration

Some participants described the effects of the integration as an “imposition”, or as the “appropriation” of the UR code system:

“I had the feeling that there was an extreme attempt to force the UR system into the one of the SR, instead of doing it the other way around or integrating both on more equal terms. It feels as if they had compulsively tried to make the first fit the second, and not really make the second fit the first...” (FG_1)

As a result, certain aspects have been lost for some FG participants:

“… the first [UR] system was a bit of a stirring - stirred me up personally. That’s completely gone now, and I think that’s a shame, because when something new comes along and it stirs things up, then it always means that it’s something important, that it may move something, or something must change. And that’s completely gone now. It doesn’t trigger so many emotions anymore, at least not for me.” (FG_1)

It was noted that both the language and content of the UR code system were lost in the merged version:

I also find it very striking that the language of tree 1 [UR system] is almost completely gone in the integrated code system, it’s directiveness, openness.” (FG_1)

## Discussion

In contrast to our initial hypothesis, the participants in all three QUAL 2 FG agreed on their observations despite their different backgrounds and positions. The differences between the two code formations of the MHB sub-teams were so apparent that they could be detected across all groups. Thereby, the participants not only identified formal differences, such as the language used or the structure of the code systems, but also more substantial disparities between the contents and focus of the codes in both systems. This was seen as positioning certain aspects of PSW implementation in the fore- and others into the background – differences that will reviewed in “Different systems of knowledge ” and discussed in relation to possible explanation in “Diverging life worlds and positionalities” sections. Furthermore, the integration process of both code systems was described by many of our FG participants as invasive, resulting in a code system that resembled primarily the SR system, and differed substantially from the one produced by the UR researchers – the reasons of which are discussed in “Epistemic struggles and structural power” and the concluding chapter sections.

### Different systems of knowledge

As described in the “Results” section and presented in Table 3, the FG participants described two distinct overall logics of the code systems – an “institutional” and “interactional” logic. By placing roles and interactive dynamics centrally, the UR code system was perceived to conceptualize PSW-implementation as more of a social process emerging from the concrete (inter)actions and relationships of and between the stakeholders involved. In contrast, the SR system was described as focusing mainly on organizational factors, thereby assigning a central responsibility for a successful PSW-implementation to structural-institutional dynamics. As demonstrated above, these differences in code formation did not emerge from different sets of primary data, but rather from the varied analytical processes of their aggregation and interpretation in the two MHB-sub-teams. Despite using the same material, the researchers of the two sub-teams placed different themes and aspects at the center of their code systems and category formations, which resulted in two distinct logics regarding what is required for the implementation of PSW.

One way of understanding these distinct logics is to think of them as implementation models that provide, at least partially, substantially different answers to the question of what is vital for the implementation of PSW. Implementation models usually encapsulate assumptions or variables regarding what is required for the implementation of any intervention [35, 36]. As a result, they build on theories of how to transform, for instance, organizational cultures, interactional dynamics, or individual beliefs and attitudes (ibid). In this way, they provide guidance on how to bring about change in an institution, justifying some actions or approaches and making others seem less urgent in the process. In relation to our two code systems, such explicit or implicit advice for action can be read in the following way: while organizational and structural adjustments are in the foreground of the SR code system, many codes of the UR system focus on the transformation of roles and interactions as indispensable prerequisites for the implementation of PSW. Thus, both code systems reflect the changes that are required to reach the goal of effective PSW-implementation, but with fundamentally different emphases in each case.

Divergent understandings of how PSWs are to be introduced in mental health care institutions as a new occupational group are common in the research field of PSW implementation: Asad et al. focus on roles and relationships between professional groups and the interactional challenges that arise from these exchanges, for example [37]. Vandervalle et al. focus on the need to transform understandings of how professional identities influence interpersonal dynamics, while also addressing organizational challenges and the necessity for change in the broader political context [21]. Byrne et al distinguish between an organizational *culture* (i.e. respect and openness during collaboration, change of attitudes) and an organizational *structure* (i.e. institutional preparation, hiring processes, equipment) [12] to implement PSW; and Ibrahim et al. make use of the CIFRc framework [38] thereby considering both the organizational culture and the attitudes of the employees during processes of PSW implementation [39].

To do justice to the main aim of this manuscript – the analysis of our collaborative processes and how they impacted the knowledge produced – only short consideration can be invested on these differential logics of implementation, their full discussion being suited for another, extensive article. To summarize, our two sub-teams produced rather dissimilar “knowledge systems” [5], i.e. systems of meaning and understanding, which addressed the implementation of PSW in rather different ways, providing for diverging foci on how to act and proceed. This raises the questions, if the two code systems may be understood as complementary, each one focusing on different levels of change, in combination creating a more comprehensive understanding of what is at stake? As mentioned in the introduction, this argument is often used in the field of collaborative research to legitimize these often cost- and time-intensive approaches. It was also proposed by some of our FG participants (Table 3). The student participants in particular described the two code systems as complementary to one another, resulting in a more complete understanding of what PSW implementation might require. As much such a conclusion is true, it falls short and silences the power relations and differential positions of the researchers involved in collaborative projects. Silencing these differences may significantly impact how practical or scientific problems, such as the one of PSW implementation, are taken-up, processed or represented, as further elaborated upon in the next section.

### Diverging life worlds and positionalities

While the FG discussions dealt extensively with the differences between the two code systems, possible explanations for these differences were less explicitly addressed. This omission was certainly due to our way of inquiring. Additionally, the complexities of possible explanations are not easily addressed in the context of a single FG. To address this limitation, in the following, we (JZ and SvP) will contribute some reflections on the potential motivations for both kinds of code formation. Thereby, we draw on the experiences we have assembled in the context of this and other collaborative projects [3, 40], as well as both positioned research approaches [41–43]. This puts us in the strange situation of writing about our teamwork as if we had not been involved. Of course, this is not the case: we were a substantial part of the processes that we now reflect on retrospectively, only somewhat from an outside position.

As demonstrated in the introduction and our results, it makes a critical difference by whom knowledge is produced [29]. Research is a social process, shaped by concrete experiences, disciplinary, professional, and personal backgrounds of the researchers involved and their material-interactional conditions [28]. So it was in our project: to start with the UR, all of them had either worked as PSWs themselves or used these services in the past. Their code formation, thus, was influenced by their experiences of having used mental health care services and of coping with recurrent situations of crises, stigmatization, or exclusion. Thus, the UR related to the research topic of PSW implementation in uniquely personal ways: they have, are, or will be using mental health care or PSW services – in some instances, also on a non-voluntary basis. This situation inescapably influenced their modes of knowledge production.

To go further: the implementation of PSW can be understood as a situation in which representatives of a population, who have or could have been, at any time, deprived of their rights of self-determination, are now expected to engage in trusting relationships with other professional groups as sovereign colleagues. Having perceived these power asymmetries themselves could be enough for the UR of our research team to know that PSW cannot be introduced in mental health care institutions without substantial changes to the relationships between users, PSWs, and other professional groups. Thus, it is not surprising that the UR of our research team focused on power-laden, interactional aspects in their coding systems. Nor is it surprising that their code system more clearly highlighted the value of implementing PSW and positioned this at the very beginning of their system. This placement suggests the UR felt the need to legitimize the idea that former users can be turned into colleagues. Knowledge production, therefore, does not take place in a vacuum, but rather can be deeply shaped by institutional positions and the life histories of those involved.

Thus, the UR knowledge was shaped by their positionalities and experiences, aligning with scientific accounts from the fields of feminist, science and technology, disability or Black studies [29, 41, 43, 44], understanding academic knowledge as “saturated with history and life worlds” [41]. Using the notion of standpoint, these scholars make clear how research designs, questions and interpretations arise from shared histories and locations in relations of power. Thus, positonalities make an enormous difference epistemically, they shape what is known and maybe possible to know about an object of interest. This is especially true for qualitative research, which often leaves more room for subjective interpretations compared to quantitative one [6]. Accordingly, a set of quality criteria is advised in this field, such as interpersonal validation, member checking, or other forms of reflexivity during a project’s design, conduct and report [45], and allowing the researchers to be self-aware and to address limitations to their perspectives or standpoints. Our collaborative proceedings and this manuscript may be understood as further means of such reflexivity, attempting at relating the differential knowledge systems to the different positionalities, they may be based upon.

This raises the question of which experiences, positions, and goals could have influenced the code formation of the SR? This question did not surface at first in our (JZ and SvP) discussions, at least not for SvP. While we talked at length about the experiences and knowledge backgrounds of the UR, it took repeated and intensive questioning by JZ to make SvP investigate the SR’ motivations for their code formation. This reminded us of other collaborative projects, in which we had focused on the positions of the UR involved, while failing to pay attention to the SR’ aspirations [10]. This inequality of attention aligns with feminist and other situated research that makes clear that positions of power are usually unmarked and unquestioned, thereby receiving less analytical consideration. This “silencing” was noted by the FG participants as well: perceiving the SR code system as “seemingly neutral” – the word “seemingly” indicating the possibility that this may not be the case – they made clear that the knowledge production of the SR is also situated. The fact that the intentions and interests behind the SR system production were more hidden begs the question, what had they been in the context of our collaboration? As much this question calls for a direct answer, it can be much better answered, after understanding what has been lost through our collaboration.

### Epistemic struggles and structural power

To provide an answer, we need to take a closer look at the steps we followed to integrate both code systems. As described, the initially planned bi-directional integration process (see “The QUAL 1 interview study”) was reduced to an unidirectional one. This decision was escorted by two major methodological decisions, taken in our full research team: 1) to take the SR system as the starting point of the integration process (instead of using both code systems or the UR one as basis), and 2) to carry out this process primarily at the level of codes (while paying less attention to the integration of categories). In preparation of this manuscript, both decisions could be reconstructed retrospectively only from fragments of our team protocols, indicating an unfortunate, rather unstructured and insufficient deliberation on the following rationales, which was part of the problem, which will be elaborated in the “Concluding thoughts” section in more detail.

The first decision was justified in our team by the mandate of the funding provider – the ImpPeer-Psy5 project had to contribute empirically grounded recommendations for the implementation of PSW – and our intentions that our results may be applicable to practice. As described above, the SR Code system followed an institutional logic that, according to our views, promised greater applicability and legibility to the funder. Second, at the time of this decision, both code systems had not been completed fully due to a limited budget of time (only three months for steps 3 and 4, Table 1), whereas the SR system was perceived to be more mature, complete, and fully structured. And third, we decided against a bidirectional integration process because this would have required time and personnel resources that we simply did not have.

When discussing these rationales in our research team, we made use of the terminology of “structural power” [6], which delineates the impact of the wider context on processes of knowledge production – in our case the context of a medical university, mental health service research, the contingencies due to the funding agency etc. To provide for an example, the academia’s neoliberal imperative to deliver utilizable outcomes may strongly shape the knowledge that is produced [46]. In particular, in health services research, there is a need to produce findings that can be efficiently translated into clinical practices [47]. In our case, factors like these, for instances, have limited our ability to focus in more detail on more elusive and complex challenges to PSW-implementation, such as attitude barriers, relationship dynamics or institutional cultures, being more difficult to operationalize than the “hard facts” of some institutional-organizational requirements.

Thus, the research context in which we work has a strong impact on the knowledge that is produced. This was also observed by some of the FG participants: some saw the integration process as “necessary to adapt” the UR system to “academic needs”. Others imagined a “compromise”-solution between both MHB sub-teams to mediate “internal and external perspectives”. Other interpretations assumed a process of cooption – a phenomenon that has been discussed widely in the fields of participatory and user/survivor research [48–50]. In cooptation, emancipatory, critical knowledge derived from more marginalized positions – in our case, the knowledge of UR – is dominated by more powerful discourses and rendered ineffective through “neutralizing”, “depoliticizing”, “absorbing”, or reducing it to “empty buzzwords” [47].

The latter possibility was discussed at length in our research team but ultimately rejected, as both decisions described were taken and accepted by all members of our research team. At the same time, we acknowledge “epistemic struggles” in our teams that, already at the time of the integration of both code systems, had cost a lot of energy and emotional labor. Thus, the decision mentioned above took place at a time when our team members were either sick or already exhausted: the first half of the project - the realization and analysis of 57 (!) interviews – had just been completed and consumed immense emotional and other resources. The time pressure resultant to the iterative (QUAL-QUAN-QUAL) design of the study, with two qualitative research phases in only three years, was immense. Moreover, the integration process of the code systems within both sub-teams had already required significant effort, as well as the negotiation of frictions across the wider team on various issue, i.e. on the question of whether the research topic should be treated affirmatively or more critically.

Thinking back – and coming to the second decision mentioned above – it is certain that any negotiation at a category level would have taken considerably more time and energy, which were no more available during the research phase. In the context of any thematic analyses, the formation of categories equals a second-order analysis [31]. Thus, categories are not only central to the structure of a code system but convey its analytical focus. A more comprehensive discussion on which focus to choose for the integrated code system would have exacerbated the already existing epistemic struggles within the MHB and the overall team. To keep our work going on a pragmatic level, and our working relationships viable, we may have tried to avoid these potential conflicts by simply subordinating most of the codes under the SR categories.

Returning to the intentions and interests of the SR code formation, a hypothesis can be offered here: that of maintaining power. Collaborative research, as any research, is bound up in broader systems and power struggles and involved forms of “epistemic politics” [51]. Against this background and the finding of the subordination or loss of UR codes and categories, we – the full research group, and particularly the subgroup of SR – were confronted with the question: what if our collaborative efforts were nothing more than a further step to stabilize the epistemic paternalism of the mental health care system or the dominant clinical research approaches? If our collaborative process – despite all good intentions – contributed to the overwriting of the UR critical positions, to what extent must this be understood as a – certainly unconscious – move to prevent the destabilization of the SR’ own position and status? And to preserve the status quo of the care and science system, on which they existentially depend? These questions were painful to many of us, even more so as they may apply to other collaborative projects that we and other researchers [3, 27] have been engaged in. They also contributed to our team decision to reconstruct the integration process in detail and to make it public it in the context of this manuscript.

## Concluding thoughts

Interestingly, this observation of epistemic politics emerged also from the reading of the transcripts by some FG participants: some assumed more power-laden dynamics of “taking over” or “overwriting” the UR’ code system by the SR researchers. Others expressed that the UR codes have been “sorted” into the “already filled shelf” of the SR code system, they mentioned a loss of the “emotional”, “stirring” of the UR system that originally had “brought momentum to the whole thing” and conveyed their “system criticism “. From these citations, it must be critically considered whether collaborative approaches ultimately serve as a token, i.e. a deliberate or unconscious strategy to conceal or legitimize hermeneutical injustice and/or to stabilize the status quo of the academic knowledge production or related fields of knowledge. Or in other words: are the usual aims or motives for collaborative research – such as giving more giving a voice to marginalized perspectives, or redressing power and knowledge inequalities [52] – possible to achieve after all?

Based on our experiences in previous projects and the ImpPeerPsy project in particular, we want to conclude by answering this question with a hesitant “yes, but”, the “but” referring to conditions that are pivotal to the field of collaborative research: first, more time and resources for complex processes of negotiation are needed, a desiderate that had been voiced frequently [53]. Second, more reflexivity is necessary on various levels. On a methodological level, we should have carried out the integration process at the level of categories, instead of rather pragmatically synthesizing their codes. This would have forced us to talk, argue, and agree on more fundamental levels of analysis and related epistemological or other underlying questions, such as elementary differences of our positionalities around the implementation of PSW. Given our limited resources, the one-day workshop within our research team was what was possible, but it was insufficient to reach this level of conversation in our team.

Further, structural power can have a particular impact when decision-making processes are insufficiently deliberate or reflexive [5]. As mentioned above, in preparation of this manuscript, we were barely able reconstruct the rationales for both methodological decisions described above. As much as the rather tensed and condensed research phase, in which these discussions took place, may explain this lack of documentation and deliberation, this had enduring consequences. Given the enormous methodological scope of both decisions, we should have invested more time and energy into the development and explications of the underlying rationales. Third, such reflexivity could have included more exchange on the interpersonal and institutional-structural context, in which these decisions have taken place. In particular, the social dynamics of collaborative knowledge production must receive reflexive prioritization, understanding their epistemic value against the background of the many and varying types of needs, interests, and epistemic privileges that the researchers bring to their team work.

To summarize, collaborative knowledge production is shaped by strucural power and is mediated by various kinds of epistemic struggles, both requiring a high degree of reflexivity during the various phases of its implementation and to contextualize its findings. If the inclusion, visibility and parity of experiential knowledge is to be increased or valued, more time and resources are needed for this reflexivity to happen. This is necessary for political, social justice and epistemic reasons. Our work and the collaborative work by other research groups demonstrate that such an extra of resources may be highly rewarding. This value is balanced by the other side of the coin: the one of epstemic politics. Which one of these two sides weighs heavier is an ethical question that is difficult to answer and subject to continuous debates. The willingness to have that debate is an essential part of this practice.

## Supplementary Information

Supplementary Material 1.

## Data Availability

No datasets were generated or analysed during the current study.
