# Supplementary material for: Difference and subordination – the epistemic struggles of collaborative knowledge production in the field of mental health
Source: Res Involv Engagem. 2025 May 13;11:46. doi: 10.1186/s40900-025-00720-4 (PMC12070662; doi:10.1186/s40900-025-00720-4)
Supplement: Supplementary file 1 — Supplementary Material 1. [file 40900_2025_720_MOESM1_ESM.docx]

**Electronic Annex**

**E1:** Overall study design and study parts of the ImpPeer-Psy 5 study

**
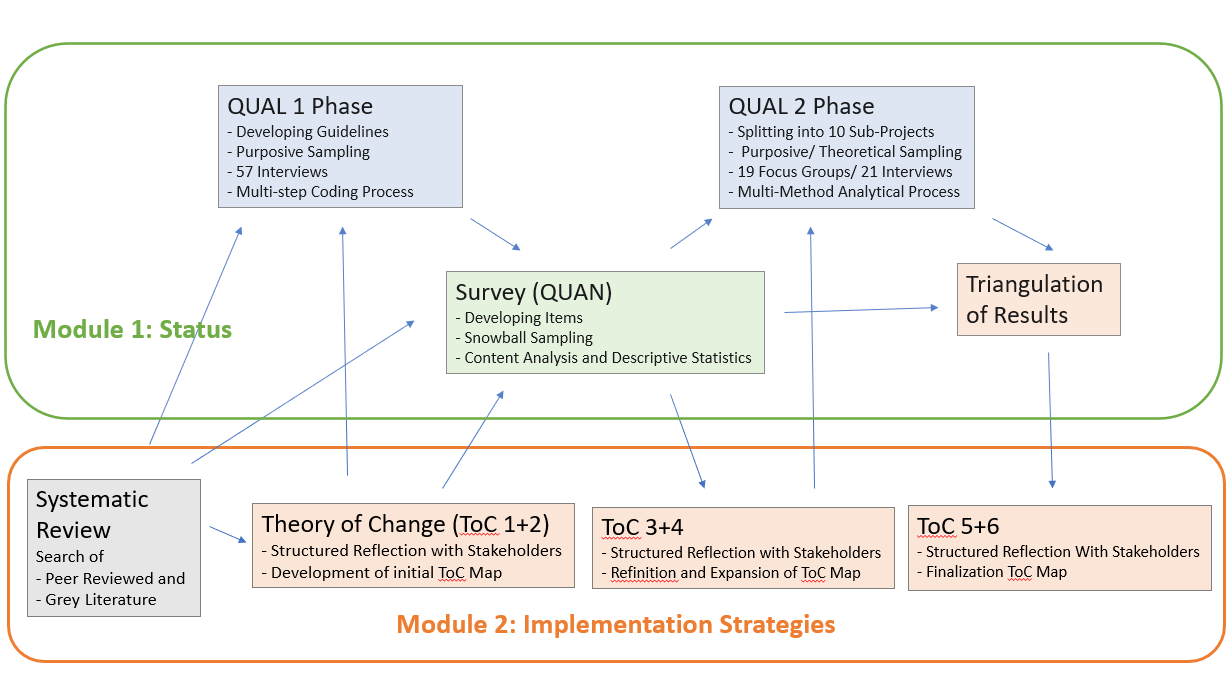
**

**E2:** GRIPP Checklist – Short Form

| **Section and topic** | **Item** | **Reported on page No** |
| --- | --- | --- |
| 1: Aim | Report the aim of PPI in the study | pp 4 & 5 |
| 2: Methods | Provide a clear description of the methods used for PPI in the study | pp 4 & 5 |
| 3: Study results | Outcomes—Report the results of PPI in the study, including both positive and negative outcomes | This is the primary topic: Whole results and discussion section |
| 4: Discussion and conclusions | Outcomes—Comment on the extent to which PPI influenced the study overall. Describe positive and negative effects | This is the primary topic: Whole results and discussion section |
| 5: Reflections/critical perspective | Comment critically on the study, reflecting on the things that went well and those that did not, so others can learn from this experience | This is the primary topic: Whole results and discussion section |

**E3**: Socio-Demographic Composition of the Population of the QUAL1 interview study

|  |  | **PSWs** | **Staff** | **User** |
| --- | --- | --- | --- | --- |
| **Gender** |  |  |  |  |
|  | f | 26 | 5 | 4 |
|  | m | 5 | 10 | 1 |
|  | diverse | 0 | 0 | 1 |
| **Age** |  |  |  |  |
|  | 20-40 | 9 | 6 | 3 |
|  | 41-60 | 21 | 11 | 2 |
|  | 61-70 | 2 | 2 | 1 |
|  | >71 | 0 | 0 | 0 |
| **Regions** |  |  |  |  |
|  | North | 10 | 5 | 1 |
|  | West | 7 | 4 | 2 |
|  | East | 9 | 5 | 3 |
|  | South | 5 | 5 | 0 |
| **Practicing PSW since** | |  |  |  |
|  | >1 Jahr | 2 |  |  |
|  | 1-3 years | 16 |  |  |
|  | 3-5 years | 6 |  |  |
|  | 5-9 years | 4 |  |  |
|  | > 9 years | 2 |  |  |
| **First contact to PSWs** | |  |  |  |
|  | 1-5 years |  | 4 | 4 |
|  | 5-10 years |  | 8 | 1 |
|  | > 10 years |  | 3 | 1 |
| **PSW qualifications** | |  |  |  |
|  | EX-IN | 27 |  |  |
|  | Other | 5 |  |  |

**E4**: Overarching research questions for the QUAL 1 interview study

| **Thematic Fields/**  **Moduls Proposal** | **Individual Requirements (A)** | **Expectations of the PSWs (B)** | **Struktural Requirements (C)** | **Tasks und Roles (D)** | **Perceived Changes (E)** | **Peserving the specifities of PSW (F)** |
| --- | --- | --- | --- | --- | --- | --- |
| **Current Practices:**  **How is PSW currently implemented in Germany?** | What competences (experience, training, skills) do the PSWs bring to their work? | What expectations do different stakeholders have of PSW? | What provisions have been made for the implementation and further development of PSW? | Which tasks are carried out by the PSWs, which roles are assumed? | How does the implementation of PSWs affect their institution and users? | What is being done in the institutions to preserve and develop the specific values, principles, and modes of operation of the PSWs? |
| **Nees and Requirements: What are the needs of PSWs and other stakeholders in terms of implementation?** | What competences (experience, training, skills) should the PSWs have? | What expectations should the stakeholders have PSW? | What requirements would be needed for the implementation and further development of PSW? | Which tasks and roles should be performed by PSWs and which roles should be taken? | Which changes should PSWs effect in the institutions? | What is needed in the facilities so that the specifities of the PSW is preserved and developed? |
| **Implementation Strategies: Which models of PB are suitable or conducive for supply in Germany?** | What competences (experience, training, skills) of the PSWs are necessary or beneficial for their work in the mental health care sector? | What are the stakeholders' expectations of PSW in the mental health care settings? | Which requirements are necessary / conducive for the implementation and further development of PSW in the mental health care sector? | Which roles and tasks are suitable for the implementation of PSW in the mental health care sector? | What changes can PSW effectuate in mental health care institutions? | What is necessary/promoting to preserve and further develop specific values, principles and modes of operation of PSW? |

Legend: PSW = Peer Support Work/ PSWs= Peer Support Workers

**E5**: Code system of the MHB sub-team of the user researchers

**Peer-User-Interaction**

**Role conflict**

Pressure to be healthy

U react negatively to peers "changing sides"

PSW as a role model

How the U is involved

**Effect of feedback from U for peers**

Feedback as a benefit for PSW

**Resources**

Finding the right tone

PSW has understood things that U still want to understand

**Characteristics**

PSW know everyday life on the ward from the U perspective

PSW are credible

Despite different symptoms, similar problems are overcome

Fewer prejudices due to own experiences

PSW with family expertise

**Effects for users**

U perceive PSW as more valuable than therapeutic/medical work

New opportunities

Developing hope for a positive outcome

Being able to get rid of anger

Learning to cope with everyday life again

More freedom because peers are there

Understanding each other well

U can be open

PSW promotes the U's autonomy

U experiences continuity

**Feelings**

Eye level

U feel taken seriously

U feel understood

Familiarity

Feeling accepted

**Communication**

PSW can understand more than other S

U know that PSW recognizes what is going on

Different conversation situations than with other S

More direct communication

**Interaction**

Can help U to understand their own situation

Conversation with experience-based expertise

PSW as contact person for special topics

Content of the support

Support for relatives

Search for resources

Empowerment

Dealing with coercive measures

Dealing with suicidality

Dealing with medication

PSW is not therapeutic/medical work

Initiating relationships

**Contact through presence**

Expectations of the U

Difficulties in fulfilling the U expectations

Where should there be PSW?

Motivation of the PSW

What peers should be able to do

Trust of the U in PSW

Effect of using experiential knowledge

**Peer-Team-Interaction**

**Starting something new together**

**Expectations of PSW**

**Attitudes/behaviors**

Openness of the S to PSW

Uncertainty about the role of the PSW

Interest of other S in the PSW

Previous experience with PSW in the team

Fears/hopes of PSW

No power of co-decision

Stigmatization

Having to face prejudices

Becoming part of a team

Fears/hopes in the team

Stress from PSW

Team concerns about the resilience of PSW

No deployment to acute wards with serious cases

Fear of contact

Uncertainty of the S

**Role of peers**

Process of developing the role of the PSW

**Special role**

PSW as mediator between S and U

PSW as advocate for U

PSW as translator

PSW as bridge builder between those affected, relatives and the clinic

PSW as bridge builder in the team

PSW as an additional joker in the team and on the ward

Peer as support for U in tense situations

PSW as critic of the treatment methods

Give criticism and feedback to professionals

PSW resists

S experience PB's experience of crisis as a hot potato

**Collaboration**

Collaboration as a process

Initial difficulties

Relationship between S and PSW

Change of perspective for S due to the presence of PSW

Different attitudes of management - team

Maintaining the specifics of the PSW

**Developments**

First experiences

Recognizing the competence of PSW

S trust in PSW

Time to develop trust

Good cooperation

Working in a team with other PSW

Joint supervision with S

Protection against excessive demands

**Changes/transformation through PB**

Change in the system

For colleagues

S gains knowledge through PSW

S learns more about the U experiences

Crisis experience of all S

Openness with regard to crisis experience of all S

Outing possible

Personal crisis experience can be included

How is PSW understood?

View of PSW

PSW own experiences

Role of PSW diagnoses for collaboration

No diagnostic limitation of PSW

Degree of transparency of peers' own experiences in the team PSW reception in the team

S handling of peers' previous experiences

PSW: deficits/resources

**Feedback on** PSW

From the team

Appreciation of PSW by colleagues

Importance of sympathy and emotions for recognition of PSW

Work becomes easier

**Conflicts**

PSW recognize prejudices of the employees towards users

Position of the PSW in the team

Critical voices against the PSW

Envy of the employees towards the PSW

Confrontation due to different positions

Procedure for communication in case of difficulties

No confrontation to avoid uncertainty among the employees

**Work organization**

Interfaces

Organization of contacts PSW – U

Public relations for PSW

Public relations for EXIN

Dissemination of information

Demand for PSW from the team

PSW responsible for certain U

PSW is included in treatment processes by colleagues

**Practical work (implementation)**

Expected task profile

Interaction in the team

Communication

Discussions about documentation

Data protection

Spontaneous exchange

Attendance at handover discussions

Attendance at morning rounds

Attendance at visits

Documentation for your own needs

Contents of your own documentation

Participation in documentation of care

Consultation hours, individual discussions

Group management

Optional tasks/task definition yourself

Freedom to design your own concepts

PSW bring in new ideas

Importance of previous professional experience/hobbies

Suggestions from peers are implemented

Differentiation with regard to the expectations of the employees

PSW actively approaches colleagues

Overlapping areas of responsibility

PSW take on tasks of other employees

Methods used together

**Structure/processes**

**Political prerequisites**

**Accessibility**

**Factors for implementation**

Structural obstacles

Regional differences in the possibilities of hiring PSW

Career/promotion opportunities for PSW

Shortage of S

High workload

Few PSW in the team

Stigmatization of PSW by the institution

Contradiction in role in the team and administration

Culture of dealing with one's own concerns

Recognition of PSW as a professional group

EX-IN job description

EX-IN image in the clinic

Financing options

PSW not billable

Lack of office space

Processes are clear

Establishment of the use of PSW

Type of use

Hour quota

Across all settings

Special setting

Same tasks as other professional groups

Co-determination/participation in committees

Aftercare for former U and relatives

Flexibility with regard to working conditions

Flexible working time arrangements

Risk of co-optation

Counteract co-optation

Loss of the special

Stress in everyday work

PSW right to have a say

Time

Lack of time for employees

Reasons for changing jobs

**Processes**

Preparation for implementation

From the institution’s side

Job description

Job interview

How did PSW come to the institution

PSW as part of an overall concept

Demand for PSW

Staff

Institution

Users

Attitude towards PSW

Superiors

Feedback discussions with management

Feedback from other employees

Management

Team

Requirements for hiring peers

Options for hiring PSW

Training of PSW

EX-IN

Induction training

Employees’ knowledge of implementation processes

Involvement of peers in the induction of new employees

Time

Preparation of teams for PSW

Support of the induction

by employees outside the team

Regular exchange

by employees within the team

Responsible person at management level

Support/implementation/responsibility for PSW

Process of the induction

Uncoordinated process of the induction

PSW supervision

Rights and obligations in everyday work

Working conditions

Space capacities

**Peers only**

**Individual potential**

Conducive to PSW

Experiential knowledge

Goals

**Proximity and distance/dangers**

Compassion

Peer must know his/her own limits

Empathize and experience this as a burden

Triggering by difficult cases

Initial difficulties in dealing with experience

**Special characteristics of the PSW role**

Importance of personality

Different roles from the perspective of the users

Skills

Easy to make contact

Knowing how something feels

Knowing how to "get out of it"

PSW trust U to take responsibility

PSW can motivate U

Sense of what U needs at the moment

PSW can assess U's situation

Being able to empathize

Being able to empathize without being burdened themselves

Compassion

Openness

**Specific methods**

Use of experiential knowledge

Required by U

U-oriented use of experiential knowledge

Degree of openness with own experiences

Specific conditions

Time

Time for individual discussions

Time to accompany outings

Time for joint activities

Time for U to get something done

PSW as a trainer in training courses for other employees

Employee observation in peer-led groups

Group management

Consultation hours

**Satisfaction/evaluation**

Personal expectations

Personal goals

Personal commitment beyond PSW

Personal satisfaction

Satisfaction with pay/classification

Resilience

Mental hygiene

Work stress

Value of training/preparation

Internships for training

Criticism of training

Evaluation of the clinic/employer

Evaluation of one's own work and conditions

Climate in the clinic/at the employer

**E6**: Code system of the MHB sub-team of the staff researchers

| **Codes Peer Support Workers** | **Definition** |
| --- | --- |
| **Motivation to become a PSW** | Why did a person become a PSW? |
| **What do PSW bring with them?** | What skills, experiences, qualifications do PSW bring with them? |
| Treatment experience |  |
| Life experience |  |
| Professional/training experience beforehand |  |
| Other experience and qualifications |  |
| **(Function) PSW training (e.g. ExIn) (incl. internships)** | Statements about PSW training (especially ExIn), what is it for, what does it bring, what could be improved? Including experiences in internships |
| Internships |  |
| Experience in internships |  |
| Potential for improvement in internships |  |
| Training/“theory part” |  |
| Experience in training/theory part |  |
| Helpful training content |  |
| Potential for improvement in training |  |
| State recognition of training |  |
| Function training (incl. internships) | What did people learn through the PSW training/internships? How did they prepare for the job as a PB*? |
| Get to know and take along forms of support |  |
| Learning to change perspectives |  |
| Being prepared/strengthened by the training |  |
| Learning that you can share about yourself |  |
| Implementation of an in-house training course for PSW | (better than implementation idea?) |
| Financing options for PSW training |  |
| Paths in | How did a PSW get their job? |
| Unsolicited application by PSW / request from PSW |  |
| Application in response to a job advertisement |  |
| Active recruitment by employers |  |
| Already been in the facility as a patient |  |
| Internship as an entry point/possibility of finding a job through an internship |  |
| "Tailwind from above" helps (only to a limited extent) | Boss/employer definitely wants PSW |
| Experiential knowledge as a prerequisite for employment |  |
| **Preparatory measures in the institution** | Statements that deal with the topic of preparations in the team for PSW; even if this did not take place |
| (No) preparation of PSW as an offer |  |
| (No) job description |  |
| (No) preparation for working with PSW |  |
| There was preparation, but it was not helpful |  |
| Team-building measures before starting work | Measures that lead to the team growing together |
| Suitable recruitment process |  |
| (No) training | Statements that deal with the topic of training; even if this has not taken place |
| Experience with (no) training |  |
| (Not) being introduced to the team |  |
| (No) fixed responsibilities for induction in the team |  |
| Potential for improvement in induction |  |
| **Work models** | Where is PSW located in the institution? How is it structurally integrated? |
| Satellite model |  |
| Peer Academy |  |
| **(Lack of) infrastructure** | Statements concerning spatial, technical, etc. working conditions |
| (No) accessibility |  |
| (No) own room/workplace |  |
| (No) access to other rooms |  |
| (No) access to the documentation system |  |
| Not being allowed to document | Somewhere else? |
| Use documentation space | Somewhere else? |
| Documentation requirements unclear/no expectations | Somewhere else? |
| **(Inappropriate) remuneration** | Statements dealing with the topic of remuneration |
| Lack of a pay group |  |
| (Alternative) financing models |  |
| No/inadequate remuneration expenses related to work activity |  |
| No longer (wanting to) work unpaid |  |
| Ideas for classification |  |
| **Consequences of a small proportion of the job** | Consequences of a part-time job for the work/ collaboration/position of the PSW in the team |
| Stress |  |
| Not being heard/invisible |  |
| Own information not getting through |  |
| Nothing from the team or patients notice |  |
| Participation in supervision and Fobi not possible |  |
| Opportunities |  |
| Small job share can protect against co-optation |  |
| Small job share can enable combination of different activities |  |
| **Structural dependencies** | Statements that describe how the work of the PB* depends on the “favor” of the other employees/the employer; concerns more the “structural/conceptual grammar” of the company |
| There are not enough positions for PB/ dependent on workplace |  |
| PSW depend on assignments from other employees in their work |  |
| (Have to) advertise themselves |  |
| Supervisor/mentor also advises on health issues (role confusion) |  |
| (Non-)fit of PSW offers to the clinic's concept |  |
| Authority to give instructions/ (location in) hierarchy tree |  |
| **Consequences of (lack of) legal framework** | The consequences of the lack of professional recognition of PSW |
| (Consequences) lack of billing option |  |
| Risk of appropriation |  |
| Consequences of lack of recognition as a professional group |  |
| Uncertainties in the role |  |
| Offers are not accessible to everyone |  |
| Professional group cannot constitute itself |  |
| Employment logic determines work content |  |
| Fixed-term/ precarious contracts |  |
| Invisibility of PSW |  |
| PSW does not exist as a category in planning |  |
| No Fobi for PSW specifically |  |
| **Work planning** | Statements concerning the planning and implementation of one's own tasks |
| Planning work together |  |
| Little independent planning possible on the part of PSW |  |
| External determination |  |
| No choice due to employment logic |  |
| Request from the employee to get (more) involved in certain tasks |  |
| PSW plan largely themselves |  |
| Create their own position/areas of activity |  |
| Learn to create their own position |  |
| Two sides of the same coin (freedom and lack of structure) |  |
| **Offers, tasks and activities** | Offers, tasks, activities of the PSW |
| Recruit new PSW/get people interested in PSW |  |
| Open consultation hours/advice |  |
| Walks |  |
| Mobilization/activation |  |
| Networking |  |
| Lectures/teaching/training |  |
| Spending time with patients |  |
| Unpermitted tasks |  |
| Games group |  |
| Empowerment group |  |
| Modify therapy concepts |  |
| Recovery work |  |
| Find out about offers |  |
| Aromatherapy |  |
| **Activities not specific to PSW** | Carrying out activities that are not specific to the PSW |
| Spontaneous stepping in for other S |  |
| Transport activities |  |
| **Goals of your own work** | Goals that the PSW define for their own work (overlaps with the following codes) |
| Change in psychiatry |  |
| Humanization of treatment |  |
| Be a role model for U |  |
| Strengthen self-awareness in employees |  |
| Self-empowerment |  |
| ... (even more...) | If we do not combine goals and competencies, there will be more to fill here |
| **Self-defined competencies/understanding of self and role** | Self-descriptions of competencies/ own understanding of the role of the PSW |
| Other/new/creative techniques and tools |  |
| Reduce facts to the essentials |  |
| Authenticity/humanity |  |
| Promote networking between U |  |
| Building bridges between U and S |  |
| Bring in knowledge from experience |  |
| Advocate/supporter of U |  |
| Bring in new/own perspectives/ideas in teams and at U |  |
| Activation/encouragement to independence and self-care |  |
| Translator/mediator between S and U |  |
| Develop goals with U |  |
| Raise awareness among S |  |
| Ensure eye level |  |
| Understanding differently |  |
| Change psychiatry/critical attitude to psychiatry/emancipatory knowledge |  |
| Needs-oriented/no agenda |  |
| Be approachable/have time/chat |  |
| Exchange of existential feelings |  |
| Break taboos |  |
| Bearer of hope |  |
| Appreciation/resource orientation when dealing with NU |  |
| Be able to listen well |  |
| Open exchange/speaking plainly |  |
| Promote destigmatization |  |
| Encourage U to be self-effective |  |
| **Attribution of characteristics of other S** | What characteristics do PB* attribute to the other S? |
| Other S have less empathy |  |
| Other employees have less patience |  |
| Other S have no experience of crises |  |
| …. |  |
| **Expectations of other S from the PSW (from the perspective of the PSW surveyed)** |  |
| Expectations of the PSW tasks |  |
| Expectations of the PSW competencies |  |
| Watching your own limits/ensuring your own stability |  |
| Unfulfillable expectations of the PSW |  |
| “Saving psychiatry” |  |
| Functionalization of PSW |  |
| … (even more)… |  |
| **Expectations of the PB from the other S/employers** | Expectations of the PSW of the other employees/S |
| Need for support to enable work |  |
| Measures to care for your own ability to work |  |
| Respecting your own limits |  |
| Co-determination in work design |  |
| …(even more)… |  |
| **(Not) being able to contribute experiential knowledge (= EK)** | How do PSW use their EW? What obstacles are there? |
| What counts as EK? |  |
| Being able to contribute your own (experiential) perspective |  |
| (No) dedicated retrieval of EK by other employees |  |
| How do PSW contribute EK? |  |
| Essentialization of EK in the team |  |
| What is EK used for/for what purpose/with what goal? |  |
| In relation to U |  |
| In relation to other S |  |
| In relation to yourself |  |
| **Benefits from experience** |  |
| Speak the same language as U | Advantages of PSW experience expertise (compared to U and within the team) and, if applicable, role in the team associated with it |
| Trust in U |  |
| Not be so easily discouraged by severe/long-lasting complaints |  |
| EK leads to a better understanding of U |  |
| EK can relieve U and S of anxiety |  |
| EK helps to question diagnoses |  |
| Icebreaker function/get better contact with U |  |
| Keep calm/know that change takes time |  |
| **Experiences of the PSW with working in a team** | PSW experiences with cooperation |
| Helplessness of the other S in dealing with PSW |  |
| (Having to) seek support for yourself |  |
| Uncertainties in dealing with the team |  |
| Having to fight your way through |  |
| (No) constructive handling of criticism from the S |  |
| New ideas/strategies are not desired |  |
| Criticism of the structures is (not) desired/no room for criticism |  |
| Other S feel threatened |  |
| Fight for authority to interpret |  |
| Intuitively understand how the PSW is doing |  |
| First contact is made together with other EG |  |
| (Not) getting involved in new things |  |
| Not being informed |  |
| Exclusion/ distance/ othering/ outsider |  |
| Different levels of acceptance |  |
| Envy/ resentment from other team members/ feeling of competition from other employees |  |
| (Lack of) contact person/ mentor |  |
| Recognition/ appreciation (from EK) |  |
| (Not) a full/ permanent part of the team |  |
| Mistrust |  |
| Having arrived/ being well integrated |  |
| Not being taken seriously/ being accused of lacking competence |  |
| (Lack of) openness/ curiosity |  |
| (Strong) commitment to PB |  |
| Disinterest/ indifference/ ignorance |  |
| “They weren’t waiting for me” |  |
| PSW caution not to exceed their competences |  |
| Devaluation in the team |  |
| Rejection in the team |  |
| Bullying |  |
| (Having to) be careful (on both sides)/ not daring to criticize PSW |  |
| Collegial cooperation/ good exchange |  |
| Personal insecurities |  |
| (perceived) competition among PSW |  |
| **Changes over time** |  |
| PSW have to prove themselves at the beginning | The self-image of the PSW /the collaboration often changes in the course of the work – codes for this can be found here |
| Uncertainties of the PSW at the beginning of the activity |  |
| Interest of the other S in PSW grows over time |  |
| PSW as a change driver/change agent |  |
| Softening of entrenched structures |  |
| Changes in attitudes (towards PSW and U) |  |
| Changes in the atmosphere |  |
| Changes in self-understanding from U to S |  |
| **Dealing with mental crises/EK in the team** | (General) handling of crisis experiences in the team, including those of other S |
| Dealing with crises of the PSW in the team |  |
| Dealing with EK/ crises of other S |  |
| Dealing with crises of the PSW among themselves |  |
| Dealing with sick leave due to mental crises in the team |  |
| PSW as a catalyst for coming-out processes |  |
| Prevention measures for crises in the team |  |
| **(None)/ (un)suitable exchange formats** | Statements on formalized exchange formats, such as supervision, intervision, etc. and on exchange with other S |
| Function of supervision, intervision etc. |  |
| Relief through exchange in the team |  |
| Little/ hardly any/ no contact with other S to exchange ideas |  |
| Informal exchange |  |
| **(No) networking of PSW among themselves** | Function and experience of networking among PSW |
| Networking within the institution |  |
| Networking across institutions |  |
| Self-organized networking |  |
| Function of networking of PSW |  |
| Networking as protection against appropriation |  |
| Networking for self-location |  |
| Networking for information exchange |  |
| **Statements that suggest position in the institution** | Try to capture those statements in the text that implicitly or explicitly make clear the position/ role of the PSW in the team; some in vivo codes here. |
| Prejudices/attributions/fears of other S |  |
| “The entertainer” |  |
| Status of a student/intern |  |
| “Pick-up and delivery service” |  |
| The one with the “weird ideas” |  |
| Having to impose one’s own perspective |  |
| (No) own access to the documentation system |  |
| “Satellite” |  |
| Advertise for oneself, have to find work |  |
| PSW have the miracle cure/ pull the cart out of the mud |  |
| Psycho-care by colleagues |  |
| “Little light”/ “last link in the chain” |  |
| Dependent position |  |
| “Order recipient” |  |
| Patient status/ I have to look after them/ they cannot cope with stress |  |
| **Stress** | (Emotional) stress/effort associated with the work and role as PSW |
| Emotional laboratory/ soul/feeling work |  |
| Emotional Availability |  |
| Being in the minority/ in the sole position in the team |  |
| Perseverance/ overwork |  |
| Working overtime |  |
| High level of initiative required |  |
| Coming into an existing team as a representative of a new professional group |  |
| No fixed place in the processes (yet) |  |
| **Tension/ division in the role/ role conflicts** | Tensions arising from the role of the PSW |
| Division in the team due to/ based on PSW |  |
| Tension in the role as part of the job/ as protection against co-optation |  |
| Sitting between two stools |  |
| Role of the PSW during coercive measures |  |
| Already been in the facility as a patient |  |
| **(Fear of) appropriation/ assimilation** | Appropriation that happens and fears of it on the part of the PSW |
| (Having to) implement professional concepts |  |
| Being confused in the role |  |
| No recognizable, independent unit |  |
| PSW as vicarious agent of psychiatry |  |
| **Prerequisites for good work of the PSW** | Ideas, thoughts, suggestions, requirements to improve the working conditions of the PSW |
| Going along during work and internship as an opportunity to try things out |  |
| Good concept for PSW, designed in a participatory manner |  |
| Individual S (key persons/ allies) as a link between PSW and team |  |
| Trust in PSW from the other S |  |
| Regulated handling of differences |  |
| Support from the institution |  |
| Clarify communication |  |
| Inform about the PSW offer |  |
| Mentor system |  |
| Peaceful coexistence |  |
| Openness/curiosity in the team |  |
| Being able to address conflicts |  |
| (Critical) attitudes towards the system can be expressed/exchanged |  |
| PSW asserts himself/acts emancipated |  |
| Receive feedback on his own work and be allowed to give it |  |
| Internship certificate contains voices from those being supervised |  |
| **Benefits of the PSW** | What changes will the PSW bring about for the psychiatric system and the PSW itself? |
| For the institution/the system |  |
| Benefits for the PSW |  |
| Stay healthy by working as a PSW |  |
| **Maintain well-being in everyday life** | How can the well-being of the PSW be maintained? |
| Self-care as a strategy for perseverance |  |
| **Experiences of the PSW with U** | What experiences do PSW have with U? |
| Rejection of PSW |  |
| Accusation of switching sides |  |
| **Counting codes** | Codes to characterize our “respondents” |
| Place of work |  |
| Working hours |  |
| Trainer training |  |
| Type of facility |  |
| Number of PSW in the institution |  |
| Number of PSW in the unit |  |
| Already been in the facility as a patient |  |

| **List of the Codes Staff** | **Definition** |
| --- | --- |
| **Training/qualifications for PSW from the perspective of the S** | Statements from S concerning PSW training |
| Experiences of the S with PSW internships |  |
| Internship as self-experience and experience with others |  |
| Various experiences as a knowledge pool |  |
| **Recruitment processes of the PSW** | Statements concerning the recruitment processes of the PSW |
| (No) job description |  |
| "Tailwind from above" is not enough |  |
| Criteria and qualifications that were relevant for the recruitment |  |
| Criteria that played a role in the selection of the place of work |  |
| Trial work/internships to get to know each other |  |
| Advantages and disadvantages of "top-down" control |  |
| Difficulties and concerns with regard to the recruitment processes undertaken |  |
| **(No) induction/preparation of the teams** | Measures that were (not) taken in the teams to prepare for PSW or to train PSW |
| Time and space to arrive |  |
| Training in the team that makes the arrival of the PSW easier (e.g. OD) |  |
| Induction plan/guide for induction |  |
| Preparatory measures |  |
| No induction necessary because PSW knows the facility |  |
| **PSW was already in the facility as a U** | Statements that deal with how to deal with it if the PSW was already in the facility as a U |
| Lock file |  |
| Specific agreements |  |
| **Remuneration/financing** | Statements that address the topic of remuneration and financing of PSW |
| There are no possibility of invoicing the PSW |  |
| Alternative/ creative ways of financing | Classification paths beyond collective bargaining law/alternative paths |
| (Suggestions for) classification | Suggestions for classification within collective bargaining law |
| Financial remuneration is inadequate/ differences in remuneration for the same work |  |
| Further qualification sought/ implemented in order to get more money |  |
| Low remuneration corresponds to the qualification level of PB* |  |
| **Consequences of a low proportion of the PSW job** | Consequences that result from a PSW only having a small share of the job |
| **Expectations of PSW by other S** | What do the other S expect from the PSW? |
| EK/ life story are shared |  |
| Representation of the affected person's perspective/ "client's ear" even in the absence of the U |  |
| Do not make yourself an advocate for the U |  |
| Implement not only therapeutic, but also everyday practical activities with the U |  |
| Want to get involved in the organization |  |
| Tact |  |
| Know your own limits/ personal responsibility for your own well-being |  |
| Reflexivity to separate your own from the other person's |  |
| Using experiential knowledge |  |
| "Building bridges"/ mediation between U and employees |  |
| "Pull the cart out of the mud" | Saving psychiatry, or in individual cases saving it now |
| **Expectations from the PSW of the other employees** | What do the PSW expect from the other S (from the S’s perspective)? |
| On Be treated “at eye level” |  |
| “Professional competence” |  |
| **Attributions/experienced competences of PSW** | What competencies do the employees attribute to the PSW, or what do they experience? |
| PSW remain calm |  |
| “Building bridges”/mediating between U and S |  |
| Authenticity |  |
| Tact/fine perception |  |
| Better understanding of the needs of the U |  |
| Being braver/feeling free from fear |  |
| Conveying hope |  |
| Building a relationship with the U |  |
| PSW bring in other perspectives |  |
| Other perspectives lead to more respect from the S towards the U |  |
| PSW are unrealistic in their expectations of change |  |
| **Tasks taken on by the PSW** | Which specific tasks are implemented/taken over by the PSW? |
| PSW offer trainings |  |
| Documentation tasks |  |
| (No) differentiation between the tasks of PSW and other S |  |
| **Workplace design** | How is the workplace designed? |
| Workplace design by PSW |  |
| Opportunities for participation |  |
| **Described effects of PSW on the other employees** | How does the work of the PSW affect the other employees? |
| Becoming aware of your own limits of understanding |  |
| Understanding more about U through PSW |  |
| Broadening of perspectives to new ways of supporting U |  |
| Preventing resignation |  |
| Contact with PSW leads to self-critical analysis/reflection of your own role |  |
| Better, more precise listening through the influence of PSW |  |
| Putting your finger on the sore spot when employees think too much about the interests of the institution (and too little about those of the U) |  |
| Catalyst of coming-out processes of other employees |  |
| PSW leads to a feeling of inferiority in relation to their own role/work |  |
| PSW encourages employees to critically reflect on their own working conditions/workloads |  |
| PSW leads to an improvement in the self-care of all employees |  |
| **Described effects of PSW on the Institution** | How does the work of the PSW impact the institution as a whole? |
| Reduction of coercion |  |
| PSW as a driver of change |  |
| PSW initiate new ways of working |  |
| **Recognition of PSW** | Recognition of the achievements of the PSW by the S |
| High respect for the life story of PSW |  |
| PSW are well trained/ respect for training |  |
| PSW as an indispensable part of the team |  |
| **Fears/ worries/ criticism from the employees** | Fears/worries regarding (working with) PSW |
| Feared effects on their own work |  |
| Concern that working with PSW is more complex |  |
| Employees expect crises to recur with PB* |  |
| Fear of too much difference/ differing positions of the PB* |  |
| Attribution of patient status |  |
| Concern that work is too emotionally stressful for PSW (emotional labor) |  |
| PSW have more freedom and flexibility in their work (envy) |  |
| PSW talk too much about themselves/ do not notice when it becomes too much |  |
| **(Risk) of being taken over** | What dangers do the employees see with regard to the appropriation of PSW? What appropriation tendencies do they perceive? |
| **Strategic development** | Measures that the employees mention to further develop PSW at the facility and in general |
| Measures to establish PSW as a professional group |  |
| Is a PSW representation number necessary? |  |
| Make yourself attractive as an employer |  |
| Networking among PSW |  |
| Self-representation of the PSW in the institution | What conditions are needed for PSW to work well? |
| Framework concept for the implementation of the PSW |  |
| **How the PSW deals with medical knowledge components** | Statements that address the handling of diagnoses/medical knowledge by the PSW, or the expectations of the employees/ PSW in relation to this topic |
| PSW increasingly use “technical language” |  |
| Medical knowledge (seems to) create security for PSW |  |
| **Prerequisites for good (co-)working of (with) the PSW** | What conditions are needed for PB* to work well? |
| Flexible deployment so that everyone benefits |  |
| Mentoring/ mentor |  |
| Employer welfare/ dealing with crises |  |
| Own space |  |
| Own KIS access |  |
| Networking of the PSW with each other |  |
| Clear management structures |  |
| Job description |  |
| Look at barriers |  |
| (No) accessibility training |  |
| Enable job changes |  |
| Openness with the other professional groups |  |
| Openness in negotiating tasks/roles |  |
| Ward management and senior physician must be on board |  |
| Cultural change is crucial for implementation |  |
| Be able to receive PSW at a low threshold |  |
| **Statements that reflect the position of the PSW in the institution hint (“between the lines”)/ Attitude towards PSW** | Statements that implicitly or explicitly allude to the cooperation between S and PSW |
| (Implicit) Hierarchy/ Othering |  |
| Submission? No criticism allowed? |  |
| De-politicization of PSW |  |
| **Feedback from the U from the perspective of the S** | How do the U experience the PSW from the perspective of the S? |
| **Counting codes** | Codes that are counted |
| Working hours  Place of Work |  |
| Trainer training |  |
| Type of institution |  |
| Number of PSW in the institution |  |
| Number of PSW in the unit |  |
| PSW already in the institution as a U |  |

**Code Tree User**

| **List of the Codes** | **Definition** |
| --- | --- |
| **(No) prior knowledge of PSW** | U did not know PSW before their first own use |
| (No) knowledge of education/training (e.g. ExIn) |  |
| **Paths to each other/in** | How did the U come to the PSW? |
| Enter a list/report to PSW for a discussion with PB* |  |
| Be informed/be introduced to each other by other employees |  |
| **(No) expectations of the PSW** | What expectations do the U have of the PSW? |
| PSW can set boundaries |  |
| Translator function/bridge building |  |
| Find solutions together |  |
| Introduce EK/give tips |  |
| Support in everyday life |  |
| Have an understanding of U |  |
| Experience is a resource and perspective |  |
| PSW should be accessible |  |
| Expected/necessary skills for PSW |  |
| **Joint activities/experienced offer** | What do PSW and U do together? What did/does the PSW offer consist of? |
| Empowerment/information about rights |  |
| Advocacy |  |
| Advice on/arrangement of other offers |  |
| Group management |  |
| Development of crisis plan |  |
| **Experience of PSW from U** | How do the U experience the PSW? Assessing, evaluative statements/feedback |
| Transparency and authenticity |  |
| Continue to be allowed to make your own decisions |  |
| Be on the side of NU/strengthen |  |
| PSW = conversations/understanding instead of meds |  |
| Always an open ear/there and approachable his |  |
| commitment to the PSW |  |
| experience knowledge how (and how) to contribute? |  |
| Personal support/ intimacy |  |
| Intensive support |  |
| Meeting as adults |  |
| Flexibility/ negotiation possible/ needs-oriented |  |
| Long-term support |  |
| Rejection/ bad experiences with PSW |  |
| **(Attributed/ experienced) skills on the part of the PSW** | What competencies do the U attribute to the PSW, or what do they experience? |
| Resource orientation/ no clinical restriction |  |
| Help with winding down/ take pressure off |  |
| “Client ear” |  |
| (Back) to self-determination and responsibility |  |
| Reading between the lines/ more direct understanding |  |
| Professional closeness (versus distance) |  |
| Less fearful/ more down to business (than e.g. PT) |  |
| Ask the right questions |  |
| Ask open questions |  |
| Authenticity |  |
| Working (or having to work) less solution-oriented |  |
| Show new ways/ examples (through EK) |  |
| Experience and specialist knowledge available |  |
| Give new impulses |  |
| Convey hope/ positive example |  |
| Different perspective based on own experiences |  |
| Be able to understand better |  |
| **Fears on the part of the U** | What concerns/fears are directed at the PSW? |
| Are PSW in Is U “stable” enough in times of crisis? |  |
| PSW may no longer be available in the future because the position is precarious |  |
| Unclear role/ mistrust by U |  |
| (Fear of) being taken over |  |
| Pressure from outside reduces quality |  |
| **Experiences teamwork between PSW & S** | How do U experience the collaboration between PSW and S? |
| Dealing with crisis experiences in the team |  |
| Experiencing PSW as a subordinate offer |  |
| (No) joint documentation |  |
| Exercising hierarchies/ decision-making power lies with other employees/ limited decision-making power of the PSW |  |
| Payment/ recognition of the PSW is too low |  |
| **Experienced/specific effects of PSW on U** | How does the work of the PSW affect the U? |
| Get users to talk |  |
| Opportunities for tandem work between PSW and other S |  |
| (More) Sustainable findings/support |  |
| Promote reflection |  |
| Being able to tell stories more/better |  |
| **Potential for change of the PSW for psychiatry** | What changes do the U hope to see in psychiatry from the PSW? |
| **Similarities and differences between relationships with PSW and relationships with friends** | What differences do U see in relationships with friends and those with PSW? |
| **(Imagined) personal gain of the PSW** | What benefits do the PSW get from the work from the U perspective? |
| **(Function) own space** |  |
| **Counting codes** | Codes that are counted |
| Place of work |  |
| Working hours |  |
| Trainer training |  |
| Type of facility |  |
| Number of PSW in the institution |  |
| Number of PSW in the unit |  |
| PSW already in the facility as a U |  |
| U can also imagine training as a PSW for themselves |  |

**E7**: Integrated code system of the QUAL 1 interview study

**1. Career entry and motivation of the PSW**

Def.: Paths into the PB, motivations for being and remaining a PSW and goals of the work

1a. Paths into it

How PSW found the position, under which conditions/circumstances

Already been in the facility as a patient

Internship as an entry point

Criteria that played a role in selecting the place of work

Experiential knowledge as a requirement for employment

Entry through application

Unsolicited application by PSW/request from PSW

Application in response to a job advertisement

Active recruitment by employers

1b. Motivation to be a PSW

Why did PB* become a PB*

Taking advantage of difficult times

Staying healthy by working as a PSW

Personal satisfaction

1c. Benefits for the PSW/ Why work is good…

Added value of the PSW for the PSW

PSW as an intermediate stage (ways out)

Qualification as a way out of peer function to jobs change

Reasons for changing jobs

PSW as a profession because nothing else is possible

1d. Goals of your own work

Goals of working as a PSW

Change in psychiatry

Humanization of treatment

Be a role model for U

Strengthening self-awareness

Personal goals

Self-empowerment

**2. What do PSW bring with them?**

Def.: previous life experiences of the PSW, both personal and professional

2a. Treatment experiences as a U

Previous experiences with treatment

2b. Life experiences

Previous experiences in everyday life

Despite different symptoms, overcome similar problems

Skills and knowledge from other areas of life

Importance of previous hobbies

2c. Previous professional/training experience

Other types of training

Additional qualifications

Therapy educational training

Previous experience/knowledge resources from other professions/work experience

Study not helpful in practice

**3. PSW training (e.g. ExIn) (incl. internships)**

Def.: Content, orientation and influence of the PSW internship/training, evaluation and potential for improvement

3a. Internships

Experiences with internships and their conditions

Experiences of the PSW in internships

Experiences of the S with PSW internships

Contact person during the internship

Support from other S during the internship

Advocates during the internship

Instructions during the internship

Deficiencies/suggestions for adjustments/suggestions for improvement in the internship

3b. Training/"theory part"

Theoretical orientation of the training

Contents of the training

Experiences during the training

Positive/helpful aspects

Not helpful aspects

Methodological aspects of training (how does training work?)

Transfer of skills

Diagnosis orientation in training

Deficiencies/suggestions for adjustments/suggestions for improvement in training

3c. Function/value of the training (including training and internships)

Concrete effects of training and internships

Getting to know and taking along forms of support

Learning to change perspectives

Internship for self- and external experience

Being prepared/strengthened by the training/preparation for practice

Learning that you can expect from/rely on something from U

Pursued intention with/own goals of peer training

Importance for your own (private) life

3d. ExIn training (specific statements on this)

Specific experience with ExIn

ExIn training viewed critically

3e. U knowledge of PSW training/training*

What U know about the training process

3f. Financing options for PSW training

How the training is financed

3g. Implementation of an in-house training for PSW

Availability of training at the workplace

4. (No) preparatory measures in the institution

Def.: Motivation and preparatory measures of the institutions to include PB*

4a. How did PSW come into the institution?

What was the reason for establishing PSW

PSW as part of an overall concept

Demand for PSW

- by staff/institution

- "tailwind" by chief doctors

- by U

Previous experience with PSW in the team

4b. (No) preparation of PSW as an offer

How the institution and staff were prepared for PSW (or not)

(No) preparation on the part of the institution

There was preparation, but it was not helpful

(No) knowledge of the staff on implementation

Helpful training/requirements in the team

Open dialogue training

(No) preparation for working with PSW

Team-building measures before starting work

(No) suitable recruitment process

Criteria and qualifications that were relevant for the recruitment

Other requirements for hiring a PSW

(No) job description

(No) interview

Disadvantages and concerns regarding the recruitment processes

Advantages and “top-down” control of the recruitment process

4c. Concrete implementation models

How/where is PSW implemented

Satellite model (PSW work in an independent unit)

“Peer Academy” (term was mentioned)

Place of deployment

Across settings

Special setting

No use of PSW on acute wards with severe cases

**5. (No) training phase**

Def.: training phase of PSW in the facility, process and evaluation

5a. Experiences with (no) induction

How the PSW are trained

Have time and space to arrive

Training plan/guide for training

(Uncoordinated) training process

(Not) being introduced to the team

(No) responsible person/fixed responsibility at (management level)

Support of the training phase of the PSW

- by employees outside the team

- by employees within the team

- regular exchange

- by other PSW

“No training necessary because PSW knows the facility”

5b. Potential for improvement of induction

How this process could be improved

**6. (Lack of) Infrastructure**

Def.: Effects of the legal and structural requirements on PSW: Working conditions, dependencies, interaction formats (e.g. supervision), networking, no recognition as a professional group, pay, low job shares

6a. Working conditions

Specific work structures

(No) accessibility

General staff shortage

Spatial (im)possibilities

(No) access to the documentation system

Workplace design

Change of workplace (not) possible

6b. Opportunities for participation in committees

To what extent can PSW get involved in political decisions (including those concerning the institution)

6c. Structural interaction formats

How PSW and other employees can exchange ideas

(No)/ (Un)suitable exchange formats

Relief for everyone through exchange in the team

Supervision, intervision etc.

Joint supervision with professionals

Supervision among PSW

Little/ hardly any/ no contact with other employees to exchange ideas

Informal/ spontaneous exchange

6d. (No) networking of PB* among themselves

(No) coordination and communication between PSW

Networking of PSW within the institution

Networking of PSW across institutions

Self-organized networking of PSW

Function of networking of PSW

Networking as protection against appropriation

Networking for self-location

Networking for information exchange

6e. Structural/institutional dependencies of PSW

How PSW* are limited by structural conditions

There are not enough positions for PSW/ dependent on workplace

Regional differences in the possibilities of hiring PB*

PSW depend on assignments from other employees in their work

(Have to) advertise themselves

Supervisor/mentor also advises on health issues (role confusion)

(Non-)fit of PB offers to the clinic's concept

6f. (No) recognition of PSW as a professional group

Side effects of (lack of) professional organization

Uncertainties in the role

PSW does not exist as a category in the planning

Offers are not accessible to all U

Consequences for the lack of recognition as a professional group

Professional group cannot constitute itself

Few PSW in the team

Employment logic determines work content

Fixed-term/precarious contracts

Invisibility of PSW

No career/promotion opportunities for PSW

Limited appreciation

6g. (Inappropriate) remuneration

How and why PSW remuneration is/cannot be appropriate

Lack of a pay group/lack of billing options

(Consequences) of lack of billing options

Alternative/creative financing models/financing options

No/inadequate remuneration Expenses related to work activity

“Low remuneration corresponds to the qualification level of the PSW

Different payment for the same work

No longer (wanting to) work underpaid

Further qualification sought/implemented due to low pay

Ideas/suggestions for classification

Satisfaction with pay/classification

6h. Consequences of a low job share

Burdens and opportunities of a low job share

Burdens

Not being heard/invisible

Your own information is not received

Not hearing anything from the team or patients

Participation in supervision and training not possible

Disadvantage due to low working hours

Opportunities

Protection against being taken over

Combination of different activities possible

Flexibility with regard to working hours possible

PSW as an option for low earners

**7. Work planning**

Def.: Identification, allocation and coordination of PSW tasks in daily work and who is responsible for this

7a. Planning work tasks together (PSW and other employees)

The possibilities for planning the work together

7b. No or little independent planning possible on the part of PSW

PSW have little decision-making power in work planning

External determination/no choice due to employment logic

EMs demand that PBs perform certain tasks (more)

7c. PSW plan largely themselves

Burdens and opportunities of independent work planning for PSW

Opportunities for independent work design/“creating your own position”

Freedom to design your own concepts

Two sides of the same coin (freedom and lack of structure)

7d. How do U get to PSW?

How is the contact between U and PSW moderated?

Enter a list for a conversation with PSW/get in touch with PSW

Be informed/introduced to each other by other S

PSW responsible for certain U

(No) diagnostic restriction in the assignment

Contact is initiated by the presence of the PSW, e.g. on the station

First contact is (not) made together with other S

**8. Offers, tasks and activities**

Def.: various tasks that PSW perform, including tasks that are not PSW tasks or that PSW are not allowed to perform

8a. Individually design activities according to individual strengths and resources Personalized activities

8b. Different tasks in different settings

Tasks are context-dependent

8c. Recruit new PSW/ interest people in PSW

Offer information to the public

Public relations for PSW/ ExIn

8d. Open consultation hours/ advice/ individual discussions

Open opportunities for discussions

Consultation hours Individual discussions

Telephone support/ consultation hours

8e. Participation/ presence in everyday clinical life

Being part of the U everyday life

During handover discussions

During morning rounds

During doctors rounds

8f. Walks

Going for a walk with U

Accompaniment when leaving the closed unit

8g. Mobilization/ activation

Lure U out of their shell

8h. Empowerment/ education about rights

Encouragement and information about rights and competencies

8i. Information about and mediation of other offers

Help with access to other services

8j. Development of a crisis plan

Development of and collaboration on a crisis plan

Individual crisis plan

Co-determined by the U

Homework for individual further work

8k. Networking

Connection with members of the U network

Support for relatives

8L. Lectures/ teaching/ training

Teaching other S about PSW

PSW as trainer in training courses for other employees

Employees in peer-led groups

8m. Spending time with patients

Spending time together in everyday life

8n. Working out goals with U

Developing goals together

8o. Searching for resources

Looking for additional resources together

8p. Group leadership

Working as a group leader

Games group

Empowerment group

8q. Modify and apply therapy concepts

Adaptation of therapy concepts

Aromatherapy

8r. Recovery work

Work with recovery concepts and practices

8s. Experience-based content and topics of support

Offer support and your own experience in difficult situations

Coercive measures

Suicidality

Medications

Addiction

8t. Documentation tasks

(No) responsibility for documentation

Not allowed to document

Documentation requirements unclear/ no expectations

Documentation obligation

Discussions about documentation

Documentation together with U cooperation

Use of documentation freedom

No need to document communication with U

Documentation for your own needs

8u. Not permitted tasks

Activities prohibited for PSW

8v. Activities not specific to PSW

Activities that PSW carry out that are not part of their role

Spontaneous stepping in for other professional groups

(No) differentiation between tasks of PSW and other employees

PSW take on tasks of other employees

Participation in documentation of care

Transport activities

Same tasks as other professional groups

Partially overlapping areas of responsibility

**9. What characterizes PSW? (experienced or attributed to oneself)**

Def.: Potentials and abilities of the PSW, attitudes, communication style, roles and role conflicts, methods used, difference to other employees - level of definition, description, especially from the perspective of the PSW

9a. Individual potentials:

Individual personality and commitment of PSW also beyond PB

Importance of the personality of the PSW

Personal commitment beyond PSW

9b. Communication/understanding:

Type of understanding, of the empathy and communication style of PSW

Reducing facts to the essentials

Conversation at eye level

PSW are credible

Understanding differently

Tact/fine perception

Empathy

Being able to empathize/NU knows that PSW recognizes what is going on

Being able to empathize without being burdened themselves

Compassion

Being able to listen well

Better communication

Different conversation situations than with other employees

Conversation easier than with doctors

More direct communication/open exchange/speaking plainly

PSW has understood things that U still wants to understand

Better understanding of U needs

Openness

Ask open questions

Ask the right questions

Find the right tone

Feeling taken seriously (as U)

Feeling accepted (as U)

9c. “Methods”:

The tools of the PSW: (Creative) methods, techniques, knowledge, networking used

Other/new/creative techniques and resources

No set methods/guidelines

Bringing in new/own perspectives/ideas to teams and to U

Promoting networking between U

Bringing in experiential knowledge (if not as a method, then see block experiential knowledge)

Activation/encouragement/empowerment

Bringing in emancipatory knowledge

Exchanging existential feelings

Resource orientation when dealing with U

Working (having to) in a less solution-oriented way

Showing new ways/examples (through experiential knowledge)

(both:) Experience and specialist knowledge available

Giving new impulses/showing possibilities

9d. Attitude/approaches

The attitude of the PSW and its effect on dealing with U and the system

Authenticity/humanity

Critical attitude towards psychiatry

Needs orientated

Fewer prejudices based on personal experiences

Breaking taboos

Appreciation when dealing with NU

PSW keep calm

Be braver/be free of fear

No clinical constriction

9e. Described roles/functions

Taken and described roles and functions of the PSW for all actors

Advocate/supporter of the U

Translator/mediator/“bridge building”

between S and U

between U and caregivers and S

between S and S

Raising awareness among S

Being approachable/having time/chatting

PSWB are the only ones who have time for conversations

Time for one-on-one conversations

Time to accompany outings

Time for joint activities

Time for U to get something done

Bearer of hope

Developing hope for a positive outcome

PSW as contact person for special topics

PSW as keeper of secrets

PSW is not therapeutic/medical work

U experiences continuity/continuous contact person

9f. Tension/split in the role/role conflicts

Where the role becomes a problem

Multiple roles from the perspective of the U

Split in the team due to/based on the PSW

Tension in the role as part of the job/as protection against being taken over

Sit on the fence

Role of the PSW during coercive measures

9g. PSW compared to other S

Other S as a comparison slide for what is special about the PSW

Other S have less empathy

Other S have less patience

Other S have no experience of crises

PSW remain more relaxed than the S

Professional closeness (versus distance)

PSW make contact more easily

9h. What is not a PSW?

What should and do not want to use PSW for.

**10. (Dealing with) experiential knowledge**

Def.: Description, use, benefits and goals of PSW experiential knowledge in daily practice, both in dealing with U and with other S, but also in general. This block is also intended to make the big term more tangible and, if necessary, to be able to differentiate it more clearly from other things. Whenever the exact term is mentioned, the example is automatically placed in this block here.

10a. What counts as experiential knowledge (=EK)?

What do the actors understand by EK, who has EK and what is EK?

PSW with family expertise

Experiences are a resource and a perspective

10b. Being able (or not) to contribute your own (experiential) perspective

Opportunities to contribute EK or the lack thereof

Conducting discussions with experiential expertise

10c. Dealing with EK within the team

How does the team/PSW deal with the potential of EK in the team?

(No) dedicated retrieval of EK by other S

Homogenization of EK in the team (differences are not seen)

Degree of transparency/openness with the peers' own experiences in the team

Dealing with EK from other professional groups

10d. How do PSW bring in EK?

Type of bringing in EK

10e. What is the aim of bringing in EK?

Aims of bringing in EK for oneself and in dealing with S and U

With regard to U

With regard to other S

With regard to oneself

10f. What can EK do? (also expands the category “What do PSW bring with them?”)

Potential of EK in practice

Different perspectives based on personal experience

Speaking the same language as U/asking the right questions

Trusting U

EK leads to greater understanding of U

EK can calm U and S down

Icebreaker function

Keeping calm/knowing that change takes time

EK helps to question diagnoses

Knowing how something feels

Knowing how to “get out of it”

**11. Expectations**

Def.: formulated (also implicit) mutual expectations

11a. Expectations of the PSW from the S and U

Expectations of the PSW in terms of their work, roles and self-care

Expectations of the PSW’s tasks

Expectations of the PSW competencies

Watching your own limits/ ensuring your own stability/ taking responsibility for your own well-being

Introducing EK/ sharing your life story

Representing the perspective of those affected even in the absence of the U

Implementing not only therapeutic activities, but also everyday practical activities with the U

Wanting to get involved in the organization

Tact/sensitivity

Reflexivity, in order not to project your own experiences onto the other person

“Building bridges”/ mediation between U and S

Finding solutions together

Having an understanding of U

PSW should be accessible

Unfulfillable expectations of the PSW

“Saving psychiatry”

Functionalization of PSW

“To sort out the mess”

11b. Expectations of the PSW tasks

PSW expectations of other S in order to be able to work well

Support for enabling work

Care measures for one's own ability to work

Respecting the limits of the PSW

Treating PSW at "eye level"

"Professional competence"

Co-determination in work design

11c. Implicit expectations

Expectations that I perceive between the lines (interpretative) - affects all actors

**12. Fears**

Def.: Fears of the individual actors (PSW, S, U). Classified here if the actor specifically expressed a concern, doubt or fear.

12a. Fears of the PSW

Fears on the part of the PSW

No co-determination

Having to face prejudices

Outing as a PSW increases stigmatization

Fear of being critically observed

(Fear of)/ (danger/risk of) appropriation/assimilation

Having to implement professional concepts

Being confused in the role

No recognizable, independent unit

PSW as an agent of psychiatry

Loss of the special

Adaptation of the peers

“Countermeasures”

Maintaining the specificity of the PSW

Counteracting appropriation

12b. Fears of the S

Fears on the part of the other professional groups

Feared effects on one's own work

Concern that working with PSW is more complex

Fear of too much difference/diverging positions of the PB*

Team's concern about the (emotional) resilience of the peers/ employees expect crises to recur with PSW

PSW talk too much about themselves/don't notice when it gets too much

PSW make themselves too strong an advocate for the U

PSW are unrealistic in their expectations of change

12c. Fears of the U

Fears on the part of the U

Are PSW "stable" enough in times of a U crisis?

PSW could no longer be available in the future because the position is precarious

Unclear role/mistrust by U

(Fear of) appropriation of the PSW by the institution

Institutional pressure reduces the quality of work

**13. Collaboration**

Def.: experienced collaboration from the individual perspectives, including mutual acceptance, challenges and dealing with crises

13a. Experienced collaboration in the team

Experienced (good or difficult) collaboration from the perspective of all team members, aspects that shape it

Uncertainties from the perspective of all team members

Uncertainties of the other employees in dealing with PSW

Helplessness of the other employees in dealing with PSW

Be careful (on both sides)

Caution on the part of the PB* not to exceed their authority

PSW shy away from confrontation in order to avoid uncertainty among the employees

Employees do not dare to criticize PSW

Criticism culture

(no) recognition/appreciation/feedback from employees

Criticism of the structures is (not) desired/ no room for criticism

(No) constructive handling of criticism on the part of the employees

Challenges

PSW have to seek support for themselves

PSW have to fight their way through

(Perceived) competition among PB*

PSW recognize prejudices of the employees towards U

Role of diagnoses for collaboration (PSW/team)

Confrontation due to different positions (PSW/Team)

Fight for authority to interpret

Exclusion/ distance/ othering/ discrimination/ bullying/

stigmatization (to be sorted out more precisely later)

Not being taken seriously/ attribution of lack of competence

Mistrust by other S

Other S feel threatened

Envy/ resentment by other team members/ feeling of competition from other S

PSW have more freedom and flexibility in their work (envy)

(Lack of) acceptance

(Lack of) openness/ curiosity on the part of the S

New ideas/strategies are not wanted

(Not) getting involved in new things

Disinterest/ indifference/ ignorance

"They didn't wait for me"

Different levels of acceptance of PSW

Different attitudes between management and team

Critical voices against PSW

Teamwork

PSW is included in treatment processes by S

(Not) a full/ permanent part of the team

Having arrived/being well integrated

(Lack of) contact person/ mentor

(Not) being informed

Good relationships

EM intuitively understand how the PSW is doing

Collegial cooperation/good exchange

(Strong) commitment to PSW on the part of the other employees

Dealing with mental crises in the team

Culture of dealing with concern

Dealing with PB crises in the team

Employees experience the PSW crisis experience as a "hot potato"

Dealing with crises of other employees

Dealing with PSW crises among themselves

Dealing with sick leave due to mental crises in the team

Preventive measures for crises in the team

13b. Collaboration between PSW and S (from the perspective of the U)

Experienced (good or difficult) collaboration from the perspective of the U, aspects that shape it

Dealing with crisis experiences in the team (here only if from the perspective of the U)

Experiencing PSW as a subordinate offer

(No) joint documentation of PSW and S

(No) decision-making power of the PSW

Exercising hierarchies

Limited decision-making power of the PSW

Payment/recognition of the PSW is too low

PSW resists

13c. Experienced collaboration between PSW and U

Experienced (good or difficult) collaboration between PSW and U, aspects that shape it

Role of diagnoses for collaboration (PSW/U)

From the perspective of the PSW

Rejection of PSW

Users react negatively to "switching sides" of the PSW

Difficulties in meeting the U expectations

Feedback from U

perceived as a benefit for PSW

From the perspective of the U (see also effects of PSW)

Personal/intensive support/intimacy

Transparency and authenticity

Meeting on an equal footing

Always an open ear/being there and approachable

Flexibility/negotiation possible/needs-oriented

PSW take their time

PSW are committed

Long-term support

Rejection/bad experiences with PSW

Similarities and differences between relationships with PSW and relationships with friends

Opportunities for tandem work between PSW and other S

**14. Collaboration as a process (arriving in the system)**

Def.: First experiences and changes in collaboration, effects of PSW on the clinic and S and on their own roles

14a. First experiences

Experiences of the PSW and employees in the initial period (first months, first year) of the PSW deployment

Initial difficulties of the PSW

PSW have to prove themselves at the beginning

Initial difficulties in dealing with empirical knowledge

Uncertainties of the PSW at the beginning of the activity

Initial difficulties of the S

Initial difficulties in dealing with empirical knowledge

14b. Interest of the other employees in PSW (grows over time)

Interest in the special features/expertise of the PSW changes among the S

Demand for PSW from the team

Work becomes easier

Becoming part of a team

14c. Climate in the clinic/at the employer

Working climate in the clinic and its changes

Changes in the atmosphere

Tailwind from above only helps to a limited extent

14d. EMs change their attitudes over time

Effect of getting to know each other on the S’

Recognizing the competence of PSW

Different perspectives lead to more respect from S towards U

S reduce their prejudices towards PSW

S change their perspective through the presence of PSW

14e. Development of the PSW role

Development (and adjustments) of the PSW role over time

**15. Stress and how to deal with it from the perspective of the PSW (only PB)** (see also OK “challenges of cooperation”)

Def.: Stress for the PSW through work and solution concepts

15a. Stress caused by the institutional conditions

Stressful effects of the structural conditions

Perseverance/overwhelm

Working overtime

High workload

High level of initiative required

Being in the minority/on the sole post in the team

15b. Overwhelm when dealing with U

Experiences and fears of being overwhelmed in relation to U

Doubts about being able to do enough for the U

Little confidence in one’s own competence from EK

Empathizing and experiencing this as a burden

Triggered by “difficult cases”

Emotional availability

15c. Joining an existing team as a representative of a new professional group

Experiences of being overwhelmed and fears of being overwhelmed in relation to the team

Pressure to be healthy

No fixed place in the processes (yet)

15d. Maintaining well-being/resilience in everyday life (of the PSW)

Strategies for dealing with stress and being overwhelmed

Self-care as a strategy for perseverance

Ensuring personal satisfaction

Psychological hygiene

Differentiating

PB* must know their own boundaries

Differentiating against attributions by the S

Differentiating with regard to the expectations of the S

Differentiating with regard to the expectations of the U

**16. Requirements for good work by the PSW/ conducive to the PSW**

Def.: Best practice of the requirements, conditions and interpersonal interaction for PSW

16a. Structural conditions/ preparation

Helpful aspects of the structural requirements

Cultural change is crucial for implementation

Good concept for PSW, designed in a participatory manner

Framework concept for the implementation of the PSW

Preparation for implementation

Mentoring system

Individual employees as a link between PSW and team

Own space

Own KIS access

Support from the institution

Employer welfare/ Dealing with crises

Clear management structures

Job description

Accessibility training/ further education

Enabling job changes

Offering internships

As an opportunity to try things out

Networking of the PSW with each other

Looking at barriers

Regulated handling of differences

Procedure for communication in the event of difficulties

Informing about the PSW offer

16b. Interpersonal, personal requirements

Helpful things to do together

Trust of the employees in PSW

Time to develop trust

Openness among the other S/ curiosity in the team

Openness in negotiating tasks/ role

Individual requirements of the PSW

PSW asserts itself/ acts emancipated

Ward manager and senior physician must be on board

Peaceful coexistence

Being able to address conflicts

(Critical) attitudes towards the system are allowed

Receiving and being able to give feedback on your own work

Feedback discussions with management

Feedback from other employees

What needs to be understood in order to be able to reach people

16c. Strategic development

Helpful things to stay (in relation to structural requirements)

Measures to establish PSW as a professional group

Is a PSW quota needed?

Making yourself attractive as an employer (must)

Self-representation of the PSW in the facility

More participatory research

**17. Experienced/perceived effects of the PSW**

Def.: experienced, attributed and perceived effects and impacts of the PSW for the institution, S and U - level of the process

17a. PSW as a driver of change

Possibilities that PSW can soften the institution and the system and initiate a change of perspective among employees and non-employees

For the institution/the system

PSW initiate new ways of working

Reduction of coercion

Softening of rigid structures

Suggestions from PSW are implemented

For the other employees

PSW as critics of the treatment methods

Suggestions from PSW are implemented

Changes in attitudes (towards PSW)

Changes in perspective among employees through the presence of PSW

Different points of view lead to more respect from employees towards non-employees

17b. PSW promote destigmatization

Reduction of the stigmatization of S in the system and institution

17c. Effects on other S (here only from the perspective of employees!)

What changes does/did the collaboration with PSW bring about for employees?

Recognition of PSW

High respect for the life story of PSW

PSW are well trained/respect for training

PSW as an indispensable part of the team

Importance of sympathy for recognition of PSW

PSW as an asset

PSW as an additional joker in the team and on the ward

Becoming aware of one's own limits of understanding

Understanding more about U through PSW

Broadening perspectives to new ways of supporting U

Preventing resignation

Contact with PSW leads to self-critical reflection of one's own role

PSW leads to a feeling of inferiority (own role/work)

Employees critically reflect on their own working conditions/workloads

PSW leads to an improvement in the self-care of all employees

Listening better, more carefully through the influence of PSW*

Pointing out when employees think too much about the interests of the institution

Catalyst for coming-out processes of other S

Openness with regard to crisis experiences of all S

Personal crisis experiences can be included

Increase in knowledge of employees through PSW

17d. Specific effects of PSW on U (here only from the perspective of the U! See also Code EK and Code experienced collaboration from the perspective of the U)

What changes for the U does/did the inclusion of PSW bring about?

PSW are on the side of the U/strengthen U

NU can continue to make its own decisions

PSW get U to talk

Conversations/understanding instead of meds

Sustainable(er) insights/support

Make people think

Help to wind down

U can get rid of anger

U perceive PSW as more valuable than therapeutic/medical work

Importance of the personality of the PSW

**18. Statements that suggest the position of the PSW in the institution (“between the lines”)/ attitude towards PSW**

Def.: In-vivo codes, “between the lines” statements and metaphors for evaluating and describing PB

“The entertainer”

Status of a student/ intern

“Pick-up and delivery service”

The one with the “weird ideas”

Having to impose one’s own perspective

“Satellite”

Advertising for oneself, having to find work

PSW have the miracle cure/ pull the cart out of the mud

Psychological support from colleagues

“Little light”/ “last link in the chain”

Dependent position

“Take orders”

Patient status/ I have to look after them/ they can’t take any pressure

Submission? No criticism allowed?

Other formulations that disparage PSW

A-politicized PSW

**19. Counting codes**

Place of work

Working hours

(EX-IN) Trainer training

Type of institution

Number of PB* in the institution

Hourly quota (h/week)

Number of PB* in the unit

Already been in the institution as a NU
